# *MIR99AHG* is a noncoding tumor suppressor gene in lung adenocarcinoma

**DOI:** 10.1038/s41419-021-03715-7

**Published:** 2021-04-30

**Authors:** Chencheng Han, Hong Li, Zhifei Ma, Guozhang Dong, Qianyun Wang, Siwei Wang, Panqi Fang, Xiang Li, Hao Chen, Tongyan Liu, Lin Xu, Jie Wang, Jun Wang, Rong Yin

**Affiliations:** 1 Department of Thoracic Surgery, Jiangsu Key Laboratory of Molecular and Translational Cancer Research, the Affiliated Cancer Hospital of Nanjing Medical University & Jiangsu Cancer Hospital & Jiangsu Institute of Cancer Research, Nanjing, China; 2 Department of Thoracic Surgery, the Third Affiliated Hospital of Soochow University, Changzhou, China; 3 Department of Thoracic Surgery, the First Affiliated Hospital of Nanjing Medical University, Nanjing, China; 4 Department of Science and technology, the Affiliated Cancer Hospital of Nanjing Medical University & Jiangsu Cancer Hospital & Jiangsu Institute of Cancer Research, Nanjing, China; 5 Biobank of Lung Cancer, Jiangsu Biobank of Clinical Resources, Nanjing, China; 6 Collaborative Innovation Center for Cancer Personalized Medicine, Nanjing Medical University, Nanjing, China

**Keywords:** Lung cancer, Macroautophagy, Long non-coding RNAs, miRNAs

## Abstract

Little is known about noncoding tumor suppressor genes. An effective way to identify these genes is by analyzing somatic copy number variation (CNV)-related noncoding genes. By integrated bioinformatics analyses of differentially expressed long noncoding RNAs (lncRNAs) and arm-level CNVs in lung adenocarcinoma (LUAD), we identified a potential antitumor gene, *MIR99AHG*, encoding lncRNA MIR99AHG as well as a miR-99a/let-7c/miR-125b2 cluster on chromosome 21q. All four of these transcripts were downregulated in LUAD tissues partly due to the copy number deletion of the *MIR99AHG* gene. Both MIR99AHG and miR-99a expression was positively correlated with the survival of LUAD patients. MIR99AHG suppressed proliferation and metastasis and promoted autophagy both in vitro and in vivo. Mechanistically, the interaction between MIR99AHG and ANXA2 could accelerate the ANXA2-induced ATG16L^+^ vesicle biogenesis, thus promoting phagophore assembly. Additionally, miR-99a targeted a well-known autophagy suppressor, mammalian target of rapamycin (mTOR), thereby synergistically promoting autophagy and postponing LUAD progression with MIR99AHG. In summary, *MIR99AHG* emerges as a noncoding tumor suppressor gene in LUAD, providing a new strategy for antitumor therapy.

## Introduction

Tumor initiation and progression is a step-by-step process driven by the accumulation of somatic genetic mutations^1^. Somatic copy number variations (CNVs) affect larger segments of the genome in cancers than other types of somatic genetic mutations^2^. Recently, an increasing number of CNV-related genes have been identified as cancer drivers^3^, including noncoding genes. Long noncoding RNAs (lncRNAs) are critical in multiple tumor biological processes^4–6^. LncRNAs located at fragile genomic sites have been identified as tumor suppressors, indicating the significance of genome breakpoints in tumor initiation and progression^7^. As arm-level CNVs might occur 30 times more often than focal CNVs when adjusting for size^8^, linking cancer-associated arm-level copy number deletion (CND) to lncRNAs might provide new insights into understanding cancer pathogenesis.

Recent studies based on chromosomal location and sequence similarity have suggested that certain lncRNAs might be processed to produce miRNAs and be closely associated with miRNAs^9,10^. For instance, lncRNA H19 generates miR-675 promoting the progression of gastric cancer^11^. Autophagy is a programmed degradation mechanism in response to environmental challenges^12^, and is now generally believed to promote the development of formed tumors and to inhibit the occurrence of early tumors^13^. The role of these noncoding RNAs in tumor autophagy needs further investigation.

Lung adenocarcinoma (LUAD) is the most prevalent histological subtype of lung cancer which has been the leading cause of cancer death worldwide^14^. Individuals with Down’s syndrome (DS) have been found to have a markedly lower incidence of lung cancer than the age-matched general population^15^. The gene-dosage effect of the extra chromosome 21 may account for this phenomenon indicating that some tumor suppressors may exist on chromosome 21^16^. Therefore, further exploration of the tumor-suppressive genes located on chromosome 21 is warranted.

Herein, we conducted an integrated analysis of lncRNA expression profiles and LUAD-specific arm-level CNV data from The Cancer Genome Atlas (TCGA)^2^, characterizing a noncoding tumor suppressor gene, *MIR99AHG* (human miR-99a host gene) in LUAD. CND of the *MIR99AHG* gene might account for the downregulation of its transcripts: lncRNA MIR99AHG and the miR-99a/let-7c/miR-125b2 cluster. Further experiments confirmed that MIR99AHG and miR-99a could suppress progression and promote autophagy in LUAD synergistically by binding ANXA2 or targeting mTOR, respectively. Combined, we provide insights into the mechanism of malignant progression of LUAD and uncovered new targets for the diagnosis and therapeutics of LUAD.

## Results

### Identification of CND-induced downregulated noncoding RNAs in LUAD

**Fig. 1:**
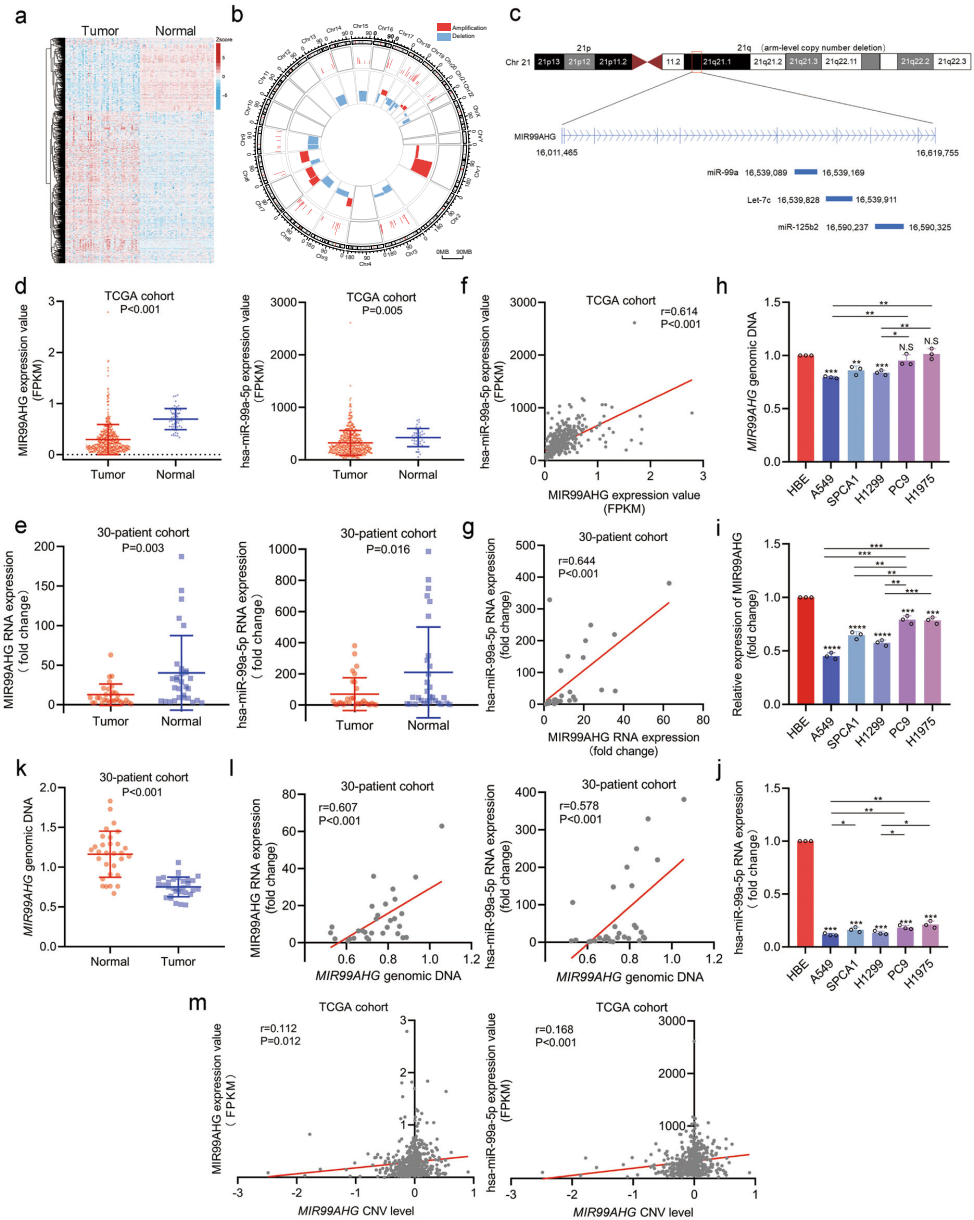
Genomic copy number deletion of *MIR99AHG* from 21q leads to the downregulation of MIR99AHG and miR-99a in LUAD. **a** Heatmap of differentially expressed lncRNAs in human LUAD tissues and normal tissues from the TCGA database. **b** Circos plot showing the association between the differentially expressed lncRNAs and the arm-level CNVs. **c** Schematic diagram of MIR99AHG family noncoding RNAs. *MIR99AHG* gene located on 21q, a copy number deletion arm, could transcribe lncRNA MIR99AHG and the miR-99a/let-7c/miR-125b2 cluster simultaneously. **d** Expression levels of MIR99AHG (*P* < 0.001) and miR-99a (*P* = 0.005) in the TCGA cohort. **e** Expression levels of MIR99AHG (*P* = 0.003) and miR-99a (*P* = 0.016) in the 30-patient cohort. **f** and **g** Correlation between expression levels of MIR99AHG and miR-99a in the TCGA cohort (*r* = 0.614, *P* < 0.001) or 30-patient cohort (*r* = 0.644, *P* < 0.001). **h** Expression level of the *MIR99AHG* gene in LUAD cell lines, with *TPTE* used as the internal control. **i** and **j** Expression levels of MIR99AHG (**i**) and miR-99a (**j**) in LUAD cells, with GAPDH and U6 used as the internal controls, respectively. **k** Expression level of the *MIR99AHG* gene in LUAD tissues and adjacent normal tissues from the 30-patient cohort (*P* < 0.001). **l** Correlation between *MIR99AHG* genomic expression level and MIR99AHG (*r* = 0.607, *P* < 0.001) or miR-99a (*r* = 0.578, *P* < 0.001) expression level in the 30-patient cohort. **m** Correlation between *MIR99AHG* genomic expression level and MIR99AHG (*r* = 0.112, *P* = 0.012) or miR-99a (*r* = 0.168, *P* < 0.001) expression level in the TCGA cohort. A two-tailed Student’s *t*-test was used for statistical analysis. *P < 0.05, **P < 0.01, ***P < 0.001, n.s. not statisticly significant. Error bars, SEM.

To investigate arm-level CND-related noncoding RNAs in LUAD, we first analyzed the lncRNA expression profiles of LUAD and nontumor tissues from the TCGA database. Compared with normal tissues, 324 lncRNAs were significantly downregulated in LUAD tissues (*P* < 0.05, fold change > 1.5; Fig. 1a). We then examined previously reported LUAD-specific arm-level CNV data^2^ and found that 124 of the 324 downregulated lncRNAs were located on the copy number deleted arm. Among them, 70 lncRNAs had a significant correlation between the expression and the amount of copy number variation (*r* > 0.1, *P* < 0.05, Fig. 1b; Supplementary Table 1). Considering that chromosome 21 may function as a tumor suppressor in LUAD^17^, we found only three downregulated lncRNAs on chromosome 21 (Supplementary Table 1). Among these, MIR99AHG caught our attention. The *MIR99AHG* gene can produce four transcripts, including lncRNA MIR99AHG itself and three derived miRNAs (miR-99a, let-7c, and miR-125b2) from q21, an arm-level CND region (Fig. 1c). We, therefore, chose the *MIR99AHG* gene for further investigation.

Based on the TCGA cohort, we found that MIR99AHG and the miR-99a/let-7c/miR-125b2 cluster were downregulated in LUAD (Fig. 1d**;** Supplementary Fig. 1a). Furthermore, the expression of MIR99AHG was positively correlated with miR-99a, let-7c, and miR-125b2 (Fig. 1f**;** Supplementary Fig. 1d). These findings were consistent with the prediction by CHIPBase and the fact that MIA99AHG is the host gene of the miR-99a/let-7c/miR-125b2 cluster (Supplementary Fig. 1c). We then performed quantitative real-time PCR (qRT-PCR) on 30 pairs of LUAD and adjacent normal tissues (30-patient cohort) for validation and obtained the same conclusion (Fig. 1e, g**;** Supplementary Fig. 1b, e). These findings confirmed that all four transcripts of the *MIR99AHG* gene were steadily downregulated in LUAD.

### Genomic CND of *MIR99AHG* accounts for the downregulation of lncRNA MIR99AHG and the miR-99a/let-7c/miR-125b2 cluster in LUAD

To explore whether the deregulation of these noncoding RNAs resulted from genomic CNV, we first assessed the expression of *MIR99AHG* genomic DNA in LUAD cell lines. Compared with the internal control *TPTE*, we observed that the genomic copy number of *MIR99AHG* was decreased in A549, SPCA1, and H1299 cells (Fig. 1h). Meanwhile, the expression of MIR99AHG and the miR-99a/let-7c/miR-125b2 cluster was markedly downregulated in LUAD cells compared with HBE cells (Fig. 1i, j; Supplementary Fig. 1f).

The expression of *MIR99AHG* genomic DNA was detected in a 30-patient cohort and we found that the genomic level of *MIR99AHG* was markedly reduced in tumor tissues compared with normal tissues (Fig. 1k). Furthermore, the genomic copy number of *MIR99AHG* was positively correlated with the expression of MIR99AHG and the miR-99a/let-7c/miR-125b2 cluster in the 30-patient cohort (Fig. 1l; Supplementary Fig. 1g). Similar findings were also obtained from the TCGA data of LUAD (Fig. 1m; Supplementary Fig. 1h). Collectively, we demonstrated that the downregulation of MIR99AHG and its derivatives in LUAD is at least partially due to the genomic CND of the *MIR99AHG* gene.

### MIR99AHG serves as a potential tumor suppressor in LUAD

**Fig. 2:**
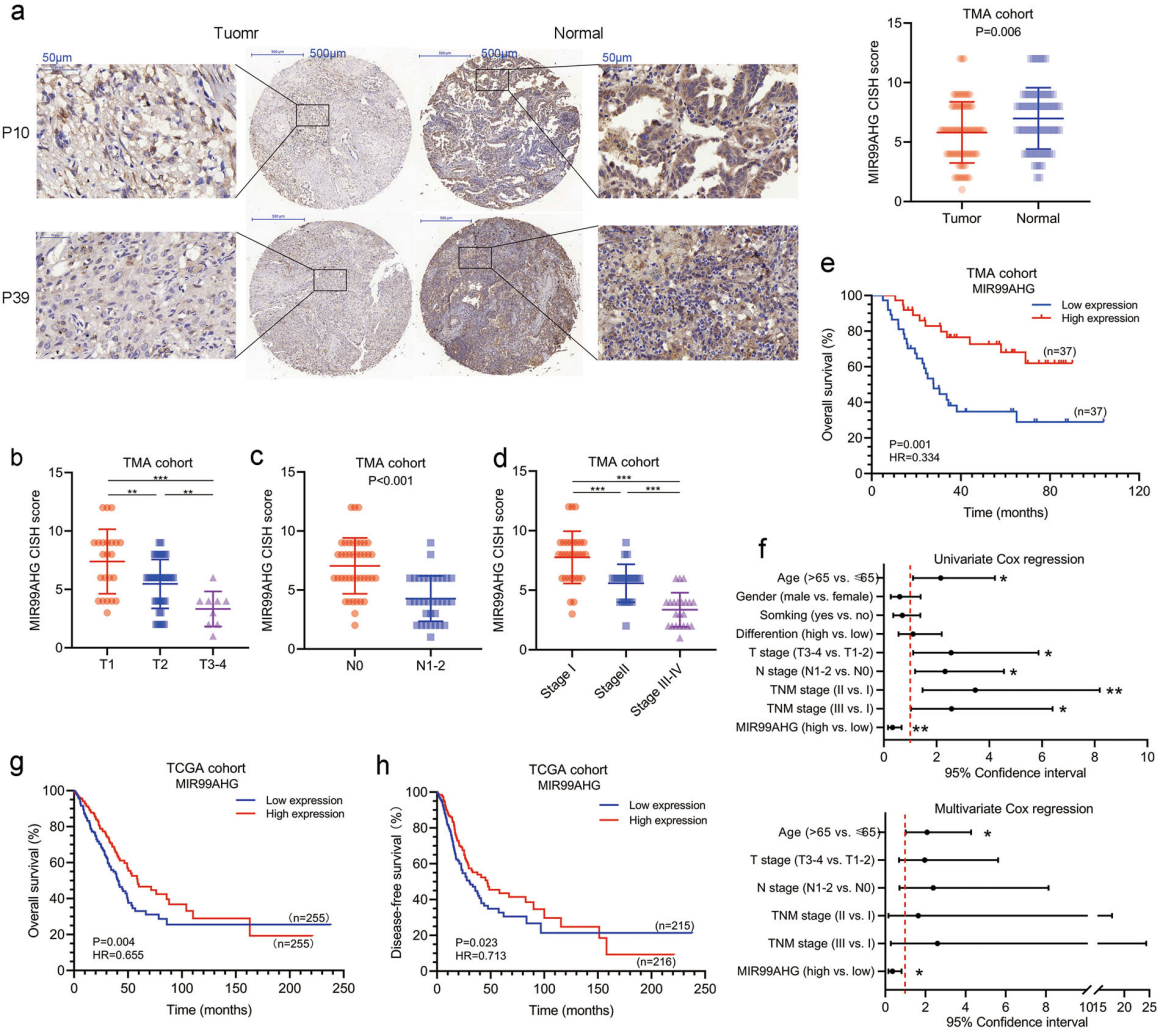
MIR99AHG is a potential tumor suppressor in LUAD. **a** MIR99AHG expression was detected by RNA chromogenic in situ hybridization (CISH) on a TMA cohort containing 74 paired LUAD tissues and adjacent normal tissues. Representative images of nontumor and tumor tissues are shown. MIR99AHG was downregulated in tumor tissues (*P* = 0.006). **b**–**d** MIR99AHG expression was downregulated in higer T stage (**b**), N stage (**c**) or TNM stage (**d**) in the TMA cohort. **e** Kaplan–Meier analysis of the overall survival in the TMA cohort based on MIR99AHG (HR = 0.334, *P* = 0.001). **f** Univariate (upper panel) and multivaraite (lower panel) analyses of overall survival in the TMA cohort by Cox regression analysis. **g**, **h** Kaplan–Meier analysis of the overall (**g** HR = 0.655, *P* = 0.004) and disease-free (**h**, HR = 0.713, *P* = 0.023) survival in the TMA cohort based on MIR99AHG. A two-tailed Student’s *t*-test was used for statistical analysis. *P < 0.05, **P < 0.01, ***P < 0.001. Error bars, SEM.

To investigate the correlation between MIR99AHG expression and clinical characteristics, we performed RNA chromogenic in situ hybridization (CISH) using a tissue microarray (TMA) containing 74 paired LUAD and adjacent normal tissues (the clinical parameters are listed in Supplementary Table 2). Compared with normal tissues, LUAD conserved lower expression of MIR99AHG (Fig. 2a). Patients with larger tumor size, lymphatic metastasis or higher TNM stage showed lower MIR99AHG expression (Fig. 2b–d) which was consistent with the data in TCGA and another 58-patient cohort (Supplementary Fig. 2a–g; Supplementary Table 3). Taken together, MIR99AHG is steadily downregulated in LUAD and negatively correlated with T, N, and TNM stages.

Survival analysis of the TMA cohort revealed that LUAD patients with higher MIA99AHG expression had longer overall survival (OS) [Fig. 2e, hazard ratio (HR) = 0.334; 95% confidence interval (CI), 0.171–0.655; *P* = 0.001]. The increased mortality risk of patients with LUAD was associated with low expression of MIR99AHG, age, tumor size, lymphatic metastasis, and TNM stage, which was shown by univariate Cox regression analysis in the TMA cohort (Fig. 2f; Supplementary Table 4). Multivariate analysis indicated that lower expression of MIR99AHG emerged as an independent prognostic factor in LUAD patients (Fig. 2f; Supplementary Table 1, HR = 0.354; 95% CI, 0.155–0.805; *P* = 0.013).

Furthermore, K–M curves exhibited similar OS and disease-free survival (DFS) tendencies in the TCGA cohort (Fig. 2g, HR = 0.655; 95% CI, 0.488–0.875; *P* = 0.004; Fig. 2h, HR = 0.713; 95% CI, 0.532–0.956; *P* = 0.023). We also validated this result in the Gene Expression Omnibus (GEO) cohort accession number GSE31210 (Supplementary Fig. 2h, *P* = 0.042). In summary, MIR99AHG may function as a potential tumor suppressor and is positively correlated with survival in LUAD.

### MIR99AHG suppresses the progression of LUAD in vitro and in vivo

**Fig. 3:**
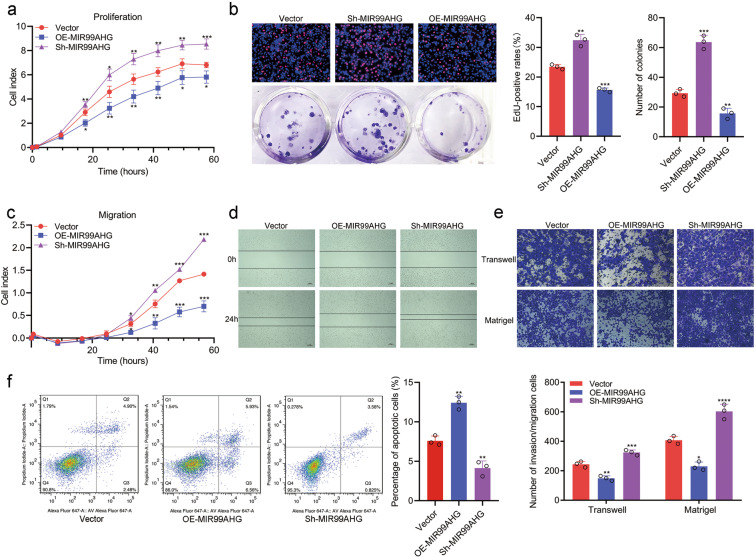
MIR99AHG suppresses proliferation, migration, and invasion of H1299 cells in vitro. **a** and **b** MIR99AHG inhibited the proliferation abilities of H1299 cells, as shown by RTCA proliferation assays (**a**), EdU assays (**b**, upper panel), and colony formation assays (**b**, lower panel). **c**–**e** MIR99AHG inhibited the migration and invasion abilities of H1299 cells, as shown by RTCA migration assays (**c**), wound-healing assays (**d**), and transwell and Matrigel assays (**e**). **f** Cell apoptosis analyses of H1299 cells transfected as indicated. Cells were harvested for Annexin-V/d PI double staining followed by flow cytometry analysis. All experiments were repeated three times with similar results and images are representative of three independent experiments. A two-tailed Student’s *t*-test was used for statistical analysis. *P < 0.05, **P < 0.01, ***P < 0.001. Error bars, SEM.

To investigate the biological function of MIR99AHG in LUAD, vectors containing the human MIR99AHG sequence were transfected into H1299 or A549 cells (Supplementary Fig. 3a). Using real-time xCELLigence analysis system (RTCA) proliferation assays, colony formation assays and 5-ethynyl2’deoxyuridine (EdU) proliferation assays, we demonstrated that exogenous MIR99AHG could inhibit the proliferation ability of LUAD cells (Fig. 3a, b; Supplementary Fig. 3c–e). Moreover, MIR99AHG suppressed the migration and invasion abilities of LUAD cells as shown by RTCA migration assays, wound-healing assays, transwell assays and Matrigel assays (Fig. 3c–e; Supplementary Fig. 3f–h). Moreover, flow cytometry analysis showed that MIR99AHG overexpression could promote the apoptosis of H1299 cells (Fig. 3f). In contrast, when silencing MIR99AHG by a more efficient short-hairpin RNA (shRNA), sh-MIR99AHG2 (Supplementary Fig. 3b), we found that the proliferation, migration, and invasion abilities were improved; however, apoptosis was impaired in H1299 cells (Fig. 3a–f). Taken together, MIR99AHG suppresses the progression of LUAD cells in vitro.

**Fig. 4:**
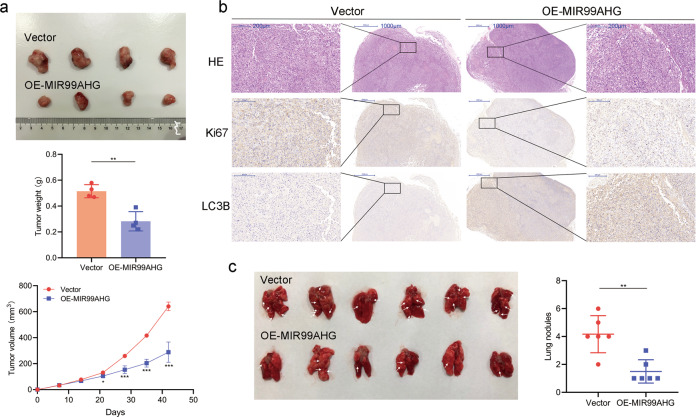
MIR99AHG inhibits tumor progression in LUAD in vivo. **a** H1299 cells transfected with the MIR99AHG overexpression plasmid or empty vector were subcutaneously transplanted into nude mice. The subcutaneous tumors at the experimental endpoint are shown. The weight and volume of subcutaneous xenograft tumors are indicated (*n* = 4). **b** IHC staining of LC3B and Ki67 performed on xenograft tumors from the nude mice. **c** H1299 cells after transfection were injected into the tail veins of nude mice. Lungs from mice in each experimental group, with the numbers of tumor nodules on lung surfaces, are shown. White arrows indicate the tumor nodules on lung surfaces. A two-tailed Student’s *t*-test was used for statistical analysis. *P < 0.05, **P < 0.01, ***P < 0.001. Error bars, SEM.

To further investigate the effects of MIR99AHG on LUAD in vivo, H1299 cells transfected with the MIR99AHG overexpression vector or control vector were transplanted into nude mice via subcutaneous or tail vein injection, respectively, which showed that overexpressing MIR99AHG suppressed LUAD growth as well as metastasis (Fig. 4a, c). Immunohistochemical (IHC) staining showed that subcutaneous tumor tissues from the enforced MIR99AHG expression group had higher expression of Ki-67 than the control group (Fig. 4b). Altogether, MIR99AHG can inhibit the progression of LUAD in vivo.

### ANXA2 serves as a binding protein of MIR99AHG

**Fig. 5:**
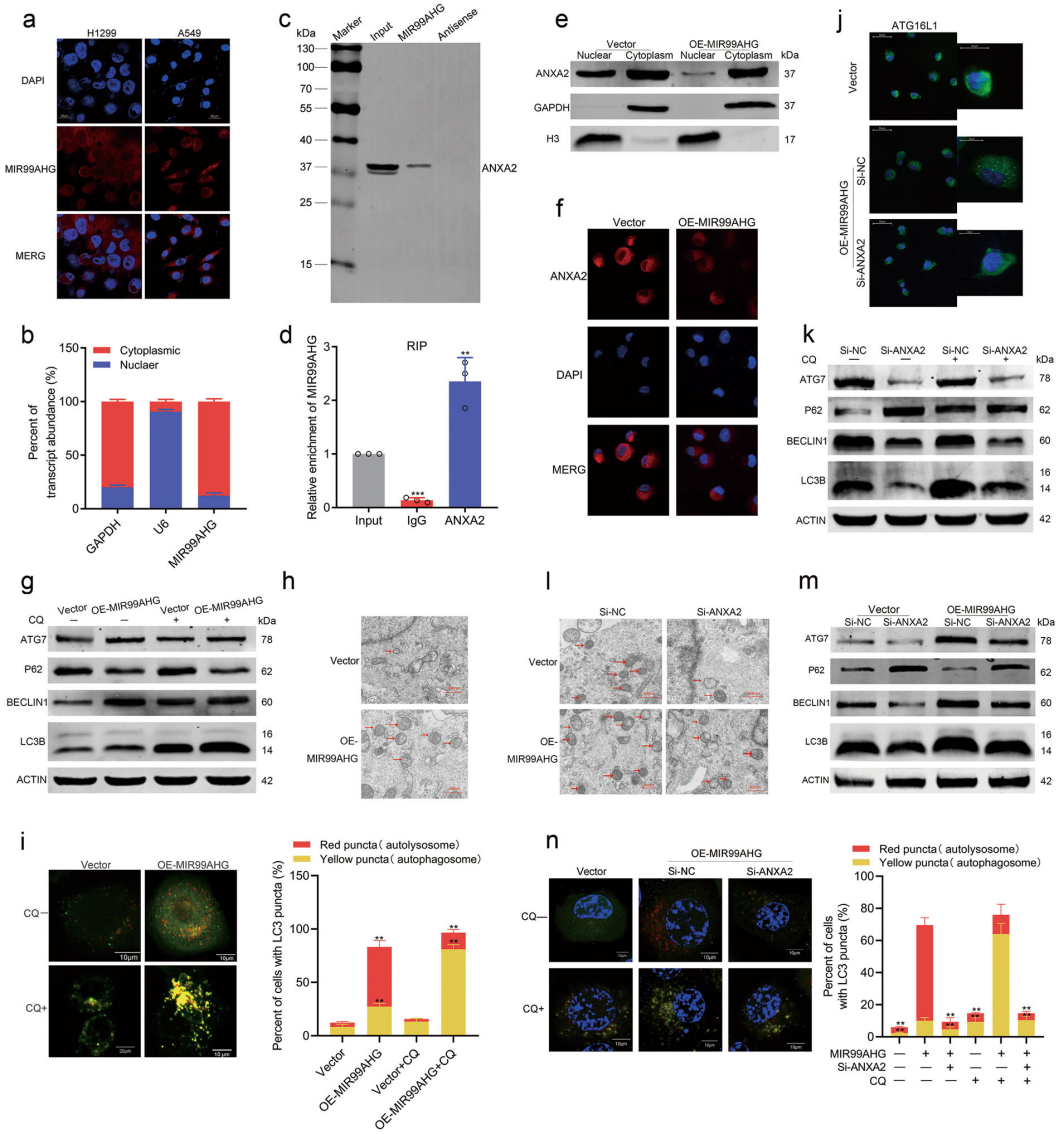
MIR99AHG binds ANXA2 and keeps ANXA2 in the cytoplasm, thus promoting autophagy of H1299 cells. **a** and **b** MIR99AHG was mainly located in the cytoplasm according to FISH (**a**) and nuclear mass separation assays (**b**). **c** In vitro transcription and pull-down assays showed that biotinylation-MIR99AHG could retrieve ANXA2. The antisense of MIR99AHG was used as a negative control. **d** RNA immunoprecipitation (RIP) experiments for ANXA2 were performed and the coprecipitated RNA was subjected to qRT-PCR for MIR99AHG. IgG was used as a negative control. **e** Subcellular fractionation of H1299 cells. Histone H3 and GAPDH served as nuclear and cytosol-specific markers, respectively. **f** Immunofluorescent analysis of changes induced by MIR99AHG overexpression in ANXA2 subcellular localization of H1299 cells. **g** Western blot (WB) analyses indicated the expression changes of autophagy-related proteins (LC3 conversion, ATG7, p62, and Beclin-1) in the indicated cells in the absence (−) or presence (+) of chloroquine treatment. **h** Ultrastructural evidence of autophagy induced by enforced MIR99AHG expression in H1299 cells. Red arrows indicate autophagosomes or autolysosomes. **i** Detection of autophagic flux with the mRFP-GFP-LC3 reporter in response to overexpression of MIR99AHG. The percentages of cells showing accumulation of yellow or red puncta were calculated. **j** Immunofluorescence assays demonstrated that MIR99AHG enhanced ATG16L^+^ vesicle formation in H1299 cells. **k**–**n** Autophagy level changes in H1299 cells induced by transfection as indicated were shown by WB assays (**k**, **l**), TEM (**m**) and autophagy flux experiments (**n**). All experiments were repeated three times with similar results and images are representative of three independent experiments. A two-tailed Student’s *t*-test was used for statistical analysis. *P < 0.05, **P < 0.01, ***P < 0.001. Error bars, SEM.

To explore the molecular mechanism underlying the biological function of MIR99AHG, we first demonstrated the subcellular localization of MIR99AHG using fluorescence in situ hybridization (FISH) analysis and nuclear mass separation assays in H1299 cells. MIR99AHG was mainly located in the cytoplasm and might play roles by binding some proteins (Fig. 5a, b). Therefore, using mass spectrometry screening and RNA pull-down assays, we validated that Annexin A2 (ANXA2) might act as a MIR99AHG-interactive molecule in H1299 cells, which was also confirmed by RNA immunoprecipitation (RIP) (Fig. 5c, d; Supplementary Fig. 4a). Further RNA pull-down assays using biotinylated truncations of MIR99AHG confirmed that the 5′ stem-loop structure of MIR99AHG was the binding domain for ANXA2 (Supplementary Fig. 4b). Taken together, ANXA2 serves as a binding protein of MIR99AHG.

### MIR99AHG promotes autophagy by binding ANXA2 in LUAD

ANXA2 is an actin-binding protein that regulates many intracellular transport events by modulating actin polymerization and participates in a variety of cellular physiological processes^18–20^. Overexpressing MIR99AHG showed no effect on the overall expression of ANXA2 at either mRNA or protein level in H1299 cells (Supplementary Fig. 4c, d). However, nuclear mass separation assays and immunofluorescence staining both confirmed that nuclear expression of ANXA2 was decreased in the H1299 cells after overexpressing MIR99AHG (Fig. 5e, f). Therefore, we speculated that the binding of MIR99AHG and ANXA2 could lead to the retention of cytoplastic ANXA2.

As several recent studies have reported that cytoplastic ANXA2 could promote autophagy^21–23^, we sorted to evaluate the effects of MIR99AHG on autophagy levels in H1299 cells. MIR99AHG overexpression changed the expression of autophagy-related proteins (upregulation of LC3B, ATG7, and Beclin-1 as well as downregulation of p62) in the presence or absence of the autophagy inhibitor, chloroquine (CQ) (Fig. 5g). Transmission electron microscopy (TEM) revealed that enforced expression of MIR99AHG increased the number of autophagic vesicles (Fig. 5h). Additionally, MIR99AHG potently induced autophagic flux to generate autophagosomes and/or autolysosomes (Fig. 5i). IHC staining showed subcutaneous tumor tissues from the enforced MIR99AHG expression group had higher expression of LC3B than the control group (Fig. 4b). Altogether, MIR99AHG could promote autophagy in H1299 cells both in vitro and in vivo.

However, MIR99AHG overexpression did not affect the mRNA expression of autophagy-related genes (ATG5, ATG7, ATG12, Beclin-1, and ULK1) in H1299 cells (Supplementary Fig. 4e). Thus, we hypothesized that MIR99AHG might promote autophagy after transcription of the autophagy-related genes. Cytoplastic ANXA2 was previously reported to promote autophagy by enhancing the biogenesis of ATG16L^+^ vesicles^21^. Interestingly, we observed increasing ATG16L^+^ vesicles in H1299 cells after overexpressing MIR99AHG (Fig. 5j). To further elucidate the effect of ANXA2 on autophagy in the H1299 cells, we designed two small-interfering RNAs (siRNAs) targeting ANXA2 and found that siRNA-2 showed a better silencing effect (Supplementary Fig. 4f, g). The autophagy-related proteins, ATG7, Beclin-1, and LC3B were all suppressed when ANXA2 was silenced (Fig. 5k). We then designed a series of rescue experiments and found that silencing ANXA2 could suppress the increase of both ATG16L^+^ vesicles and autophagy level induced by overexpressing MIR99AHG in the H1299 cells (Fig. 5j, l–n). Moreover, the MIR99AHG mutant (Mut-4) lacking the predicted ANXA2-binding domain had no effect on the autophagy level of H1299 cells, which further verified that MIR99AHG promoted autophagy by binding ANXA2 (Supplementary Fig. 4h–j). In sum, MIR99AHG can bind ANXA2, increase its cytoplasmic localization and promote ATG16L^+^ vesicle formation, thus promoting autophagy in LUAD cells.

### miR-99a acts as another 21q CND associated tumor suppressor in LUAD

**Fig. 6:**
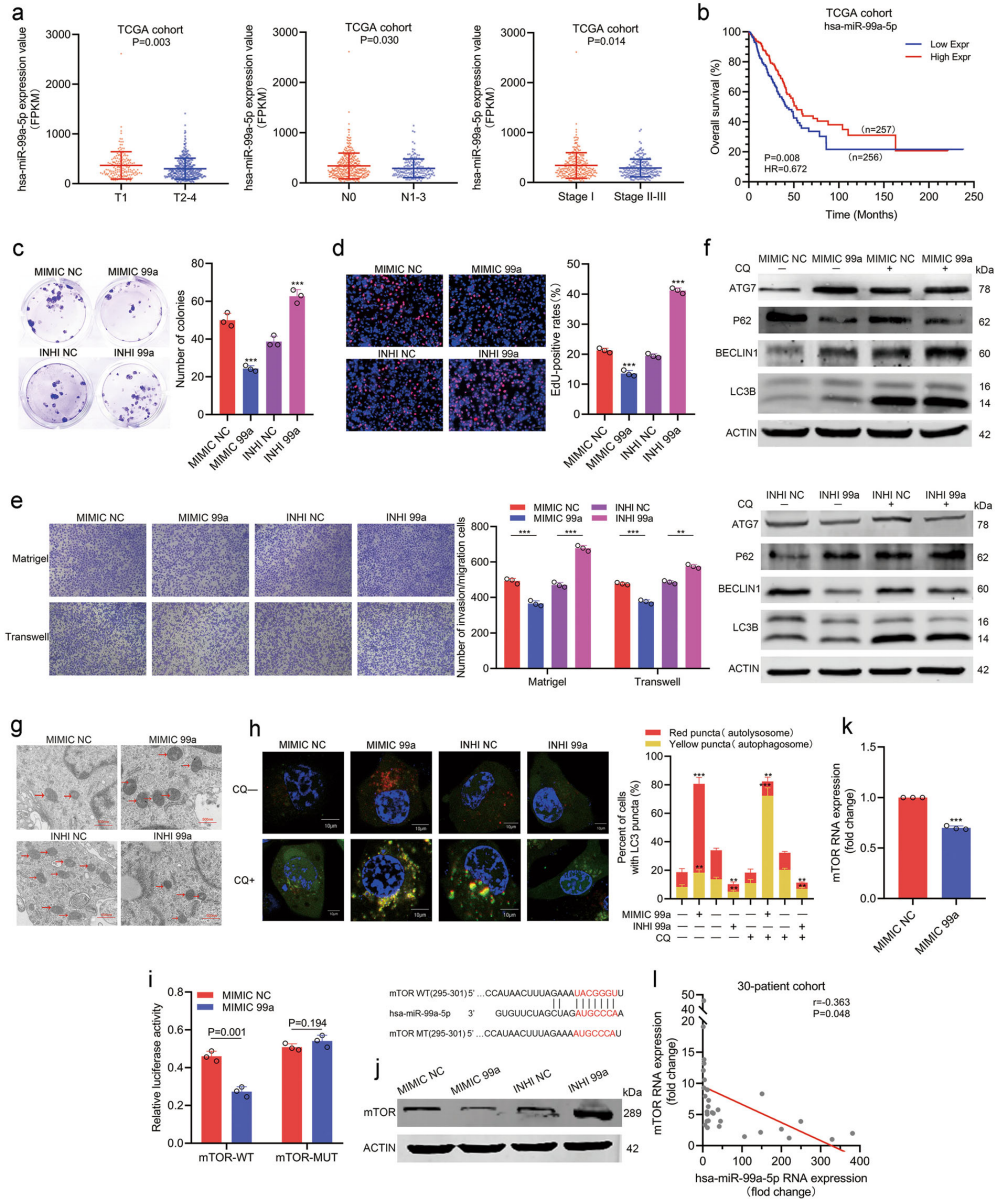
miR-99a restrains progression and promotes autophagy by targeting mTOR. **a** miR-99a expression was downregulated at higher T stage (left panel, *P* = 0.003), N stage (middle panel, *P* = 0.030) or TNM stage (right panel, *P* = 0.014) in the TCGA cohort. **b** Kaplan–Meier analysis of the overall survival in the TCGA cohort based on miR-99a (HR = 0.672, *P* = 0.008). **c**, **d** miR-99a inhibited the proliferation ability of H1299 cells, as shown by colony formation assays (**c**) and EdU assays (**d**). **e** miR-99a inhibited the migration and invasion abilities of H1299 cells, as shown by transwell and Matrigel assays. **f**–**h** miR-99a promoted autophagy in H1299 cells, as shown by WB assays (**f**), TEM (**g**), and autophagic flux (**h**). **i** The schematic diagram of the binding site of mTOR and miR-99a which was predicted by TargetScan (http://www.targetscan.org/vert_72/), and the schematic diagram of wild type or mutant mTOR sequence used for dual luciferase reporter assays. Dual luciferase reporter assays indicated that miR-99a directly binds to the 3’-UTR of mTOR (*P* = 0.001). **j** Western blot analysis indicated the change in mTOR expression level in H1299 cells transfected with miR-99a mimics or inhibitors. **k** qRT-PCR showed that miR-99a overexpression decreased the mRNA expression of mTOR in H1299 cells (*P* < 0.001). **l** Correlation between miR-99a expression and mTOR expression in the 30-patient cohort (*r* = −0.363, *P* = 0.048). All experiments were repeated three times with similar results and images are representative of three independent experiments. A two-tailed Student’s *t*-test was used for statistical analysis. *P < 0.05, **P < 0.01, ***P < 0.001, n.s. not statistically significant. Error bars, SEM.

Considering that 21q CND also leads to decreased expression of the miR-99a/let-7c/miR-125b2 cluster in LUAD, we further analyzed the association between these three miRNAs and clinicopathological features. In the TCGA LUAD cohort, we found that the expression of miR-99a and let-7c rather than miR-125b2 was decreased at higher T or TNM stages (Fig. 6a left, right panels; Supplementary Fig. 5a, c). Among the three miRNAs, only miR-99a was downregulated in patients with lymphatic metastasis (Fig. 6a middle panel; Supplementary Fig. 5b). Meanwhile, K–M survival analyses showed that patients with higher miR-99a expression had a longer OS (Fig. 6b, HR = 0.672; 95% CI, 0.501–0.902; *P* = 0.008). However, we did not observe a significant association between let-7c/miR-125b2 expression and OS (Supplementary Fig. 5d). Taken together, in addition to MIR99AHG, miR-99a, rather than let-7c/miR-125b2, might serve as another 21q CND associated tumor suppressor in LUAD.

### miR-99a promotes autophagy and inhibits the progression of LUAD by targeting mTOR

Several studies pointed out that producing embedded miRNAs would be a major role for certain lncRNAs. Furthermore, overexpressing MIR99AHG significantly increased the expression levels of miR-99a, let-7c, and miR-125b2 in H1299 cells, although the underlying mechanism remains further investigated (Supplementary Fig. 6a). Hence, we speculated that MIR99AHG may exert its biological function at least partially via the miR-99a/let-7c/miR-125b2 cluster. Mimics for miR-99a, let-7c, and miR-125b2 whose efficiency was validated were utilized to investigate the biological functions of the three miRNAs (Supplementary Fig. 6b). Using RTCA proliferation assays, we found that miR-99a and let-7c rather than miR-125b2 significantly inhibited the proliferation ability of H1299 cells (Supplementary Fig. 6c), which was consistent with our previous finding in the TCGA data. Only miR-99a was found to promote autophagy in H1299 cells by Western blotting assays, while let-7c and miR-125b2 did not (Fig. 6f; Supplementary Fig. 6d). Therefore, miR-99a was investigated in subsequent studies.

To further verify the biological function of miR-99a, we mimicked or inhibited miR-99a in H1299 cells. The results showed that miR-99a mimics could decrease the proliferation, migration, and invasion abilities of H1299 cells (Fig. 6c–e; Supplementary Fig. 6e, f), which was consistent in A549 cells (Supplementary Fig. 6g–m). Furthermore, Western blot, TEM, and autophagic flux experiments showed that miR-99a could significantly improve the autophagic level of H1299 cells (Fig. 6f–h). Mammalian target of rapamycin (mTOR), an autophagy inhibitor, has been reported previously as a potential downstream target of miR-99a^24,25^. To validate the direct interaction between miR-99a and mTOR, the wild-type or mutant 3′ untranslated region (3′-UTR) of mTOR was cloned into luciferase reporter vectors. Luciferase reporter assays showed that miR-99a could remarkably decrease luciferase activity in wild-type H1299 cells, while cells transfected with mutant vectors showed no significance (Fig. 6i). In addition, ectopic expression of miR-99a decreased mTOR expression at both the protein and mRNA levels, whereas miR-99a inhibitors increased mTOR protein levels (Fig. 6j, k). Finally, the mRNA level of mTOR was negatively correlated with miR-99a in our 30-patient cohort (Fig. 6l). Taken together, miR-99a can promote autophagy and suppress the progression of LUAD by targeting mTOR.

### miR-99a synergistically promotes autophagy and inhibits LUAD progression with MIR99AHG

**Fig. 7:**
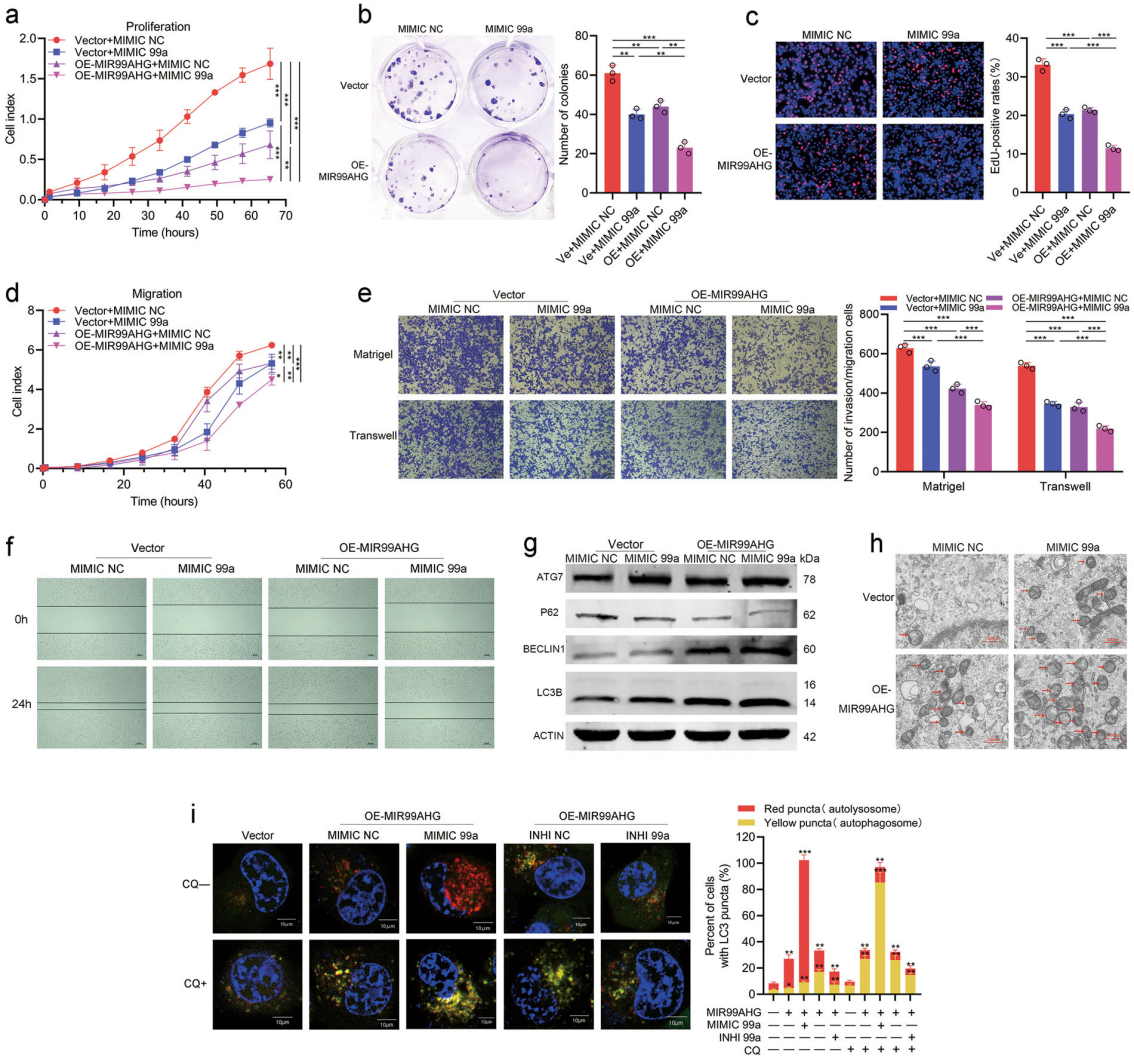
miR-99a cooperates with MIR99AHG to exert biological effects. **a**–**c** Proliferation ability of transfected H1299 cells as indicated is shown by RTCA proliferation assays (**a**), colony formation assays (**b**) and EdU assays (**c**). **d**–**f** Migration and invasion ability of indicated H1299 cells is shown by RTCA migration assays (**d**), transwell and Matrigel assays (**e**), and wound-healing assays (**f**). **g**–**i** Autophagy level of the indicated H1299 cells is shown by WB assays (**g**), TEM (**h**), and autophagic flux experiments (**i**). All experiments were repeated three times with similar results and images are representative of three independent experiments. A two-tailed Student’s *t*-test was used for statistical analysis. *P < 0.05, **P < 0.01, ***P < 0.001. Error bars, SEM.

To validate whether MIR99AHG could exert biological functions via miR-99a, we designed rescue experiments using miR-99a inhibitors and found that miR-99a inhibition could partially rescue the proliferation, migration, and invasion suppression induced by MIR99AHG in H1299 cells (Supplementary Fig. 7a–f). Furthermore, the MIR99AHG-induced increase in the autophagic level of H1299 cells could also be partly reversed by miR-99a inhibitors (Supplementary Fig. 7g, h; Fig. 7i). Thus, the role of MIR99AHG in inhibiting the progression and promoting autophagy of LUAD was mediated in part by miR-99a. On the other hand, the combination of MIR99AHG overexpression and miR-99a mimics showed a stronger effect at inhibiting proliferation, migration, and invasion abilities as well as promoting autophagy of H1299 cells compared with overexpressing MIR99AHG or mimicking miR-99a alone (Fig. 7a–i). Altogether, miR-99a synergistically promotes autophagy and inhibits the progression of LUAD with MIR99AHG.

## Discussion

**Fig. 8:**
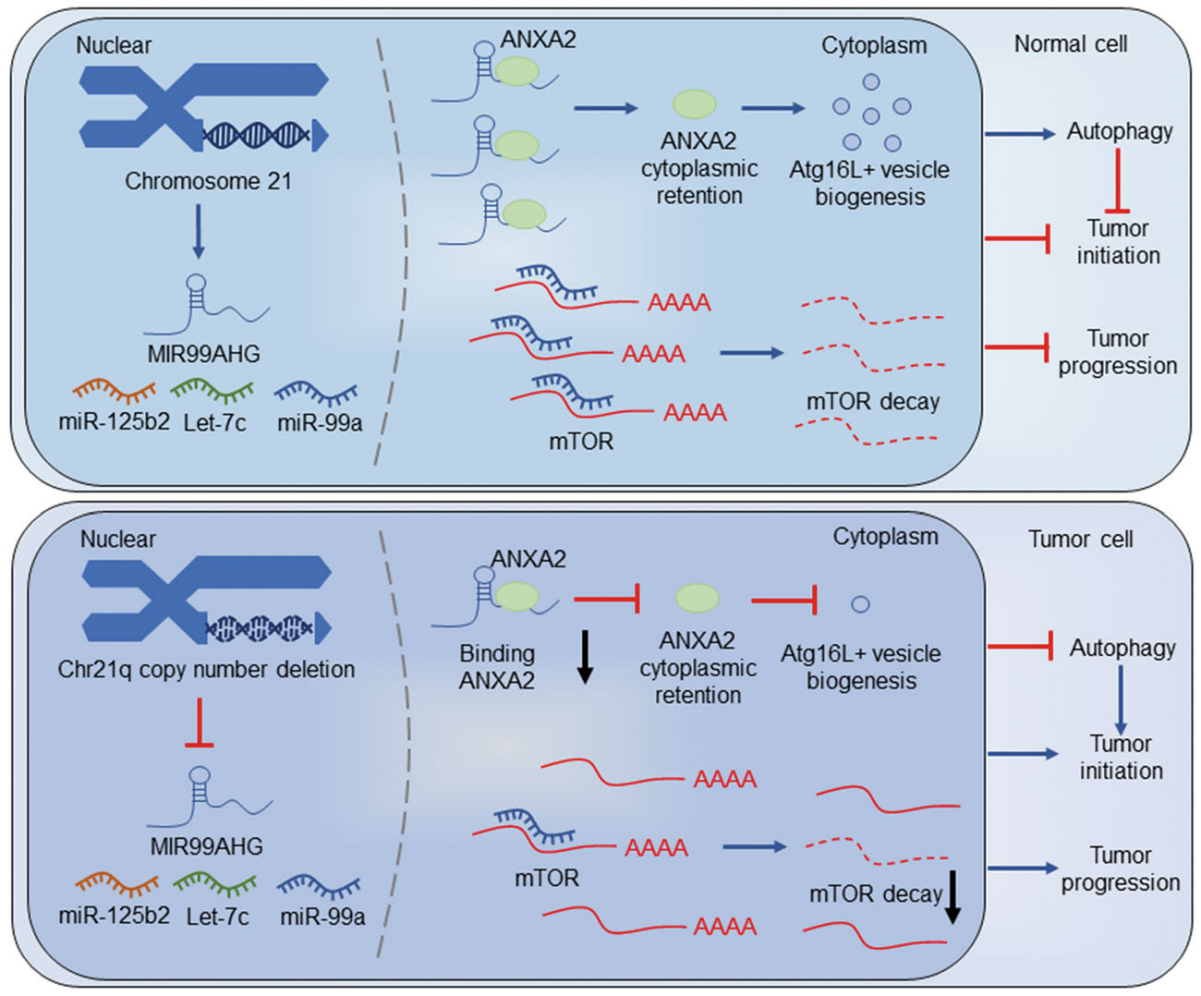
Schematic diagram of how *MIR99AHG* promotes autophagy and inhibits the progression of LUAD. In tumor cells, copy number deletion of 21q leads to the low expression of MIR99AHG and the miR-99a/let-7c/miR-125b2 cluster. MIR99AHG binds to ANXA2 to sustain cytoplasmic retention of ANXA2 which accelerates the biogenesis of ATG16L^+^ vesicles, and MIR99AHG-derived miR-99a targets mTOR, thus promoting autophagy and repressing the progression of LUAD. MIR99AHG/miR-99a-mediated autophagy enhanced the inhibition effect of these antitumor genes on the LUAD biogenesis, while loss of the autophagy promoted the tumor initiation induced by deficiency of these tumor suppressors.

In this study, *MIR99AHG* was characterized as a potential noncoding tumor suppressor gene in LUAD. Four noncoding RNAs transcribed from *MIR99AHG* (lncRNA MIR99AHG and miR-99a/let-7c/miR-125b2 cluster) were downregulated due to the CND of chromosome 21q in LUAD. MIR99AHG promoted autophagy and suppressed LUAD progression by regulating ANXA2 induced ATG16L^+^ vesicle biogenesis as well as deriving miR-99a to target mTOR (Fig. 8).

Genomic variation data has been widely used to identify oncogenic drivers in cancer. Several well-known proto-oncogenic genes have been shown to be driven by CNVs^26–28^. Compared to protein-coding genes, noncoding genes lack hotspot point mutations, while structural variations, including CNVs, are thought to contribute more to noncoding drivers^7^. Cancer drivers of lncRNA can be distinguished from passengers by lncRNA expression profile-matched clinical outcome and CNV data^29,30^, such as FAL1^31^. Besides the oncogenes, the deficiency of tumor suppressors induced by genomic CND also might be a driving factor of tumor initiation and progression^32^. Herein, we integrated the lncRNA expression profile and arm-level CNV data of LUAD from the TCGA database and identified three downregulated lncRNAs on the chromosome 21q, a CND arm (Supplementary Table 1). Besides MIR99AHG, the other two downregulated lncRNAs—LINC00315 and LINC01671—both had no correlation with overall survival in the TCGA cohort (Supplementary Fig. 2i, j). Therefore, we chose MIR99AHG for further study.

MIR99AHG is a polycistronic miRNA host gene encoding three intronic miRNAs—miR-99a, let-7c, and miR-125b2. Recently, numerous studies focused on the relationship between such polycistronic lncRNAs and their derived miRNAs. For instance, MIR100HG was reported to generate miR-100 and miR-125b to mediate cetuximab resistance^33^, and MIR22HG could act as a tumor suppressor partially by deriving miR-22^34^. Furthermore, miRNAs may serve as partners or antagonists of their host genes by functionally targeting genes associated with host genes^35^.

MIR99AHG was first reported as an oncogene in acute megakaryoblastic leukemia^36^, and later found to be involved in the formation of cellular structures in head and neck squamous cell carcinoma^37^. A recent study suggested that MIR99AHG promoted gastric cancer^38^. In addition, miR-99a, let-7c, and miR-125b2 all emerged as suppressors in several solid tumors^39–41^, while oncogene in leukemia^36^, which was consistent with the effect of chromosome 21 in DS on promoting childhood leukemia and suppressing solid tumors^15^. In this study, we found that MIR99AHG and its three derivatives were all downregulated in LUAD. Expression of MIR99AHG was positively correlated with miR-99a/let-7c/miR-125b2 cluster. MiR-99a served as a partner of the host gene in promoting autophagy and suppressing progression of LUAD. Let-7c could only inhibit progression of tumor cells, while miR-125b2 showed no functional role. Altogether, *MIR99AHG* served as a tumor suppressor gene mainly through the synergistic biological function of MIR99AHG and miR-99a.

Overexpressing MIR99AHG in H1299 cells increased the expression of miR-99a/let-7c/miR-125b2 cluster (Supplementary Fig. 6a). As the miR-99a, let-7c, and miR-125b2 are all intronic miRNAs, which could not be produced by mature lncRNA directly, we speculated that MIR99AHG might play a role in the biogenesis of the miR-99a/let-7c/miR-125b2 cluster. Reviewing miRNA biogenesis, we found that Pol II, Drosha, Dicer, and RISC are vital in producing miRNAs^42^. Several lncRNAs have been confirmed to promote or inhibit miRNA biogenesis by affecting the role of Drosha or Dicer^43–45^. As MIR99AHG mainly located in the cytoplasm, we speculated that Dicer or RISC might be a key regulation step affected by MIR99AHG. However, further studies are needed to prove this hypothesis.

Autophagy, a highly conserved basic catabolism pathway in eukaryotes, can be used to obtain nutrients as a survival mechanism by tumor cells under adverse conditions. On the other hand, autophagy can also inhibit tumor biogenesis, and the absence of autophagy may lead to malignant transformation^13^. Several studies have reported the inhibition effect of genes promoting autophagy on tumor initiation. For example, CK1α could inhibit tumor growth by inducing autophagy and inhibition of CK1α-induced autophagy could help to initiate tumorigenesis by cooperating with oncogenic HRAS^V12^^46^. We proposed a hypothesis here that MIR99AHG/miR-99a-induced autophagy might enhance the suppression effect of these antitumor genes on LUAD tumorigenesis, similar to CK1α. Furthermore, due to the CND of *MIR99AHG* gene in LUAD, the loss of autophagy induced by MIR99AHG/miR-99a might promote the tumor initiation (Fig. 8). However, the effect of autophagy induced by MIR99AHG/miR-99a on inhibiting tumor progression remains to be further studied.

ANXA2 was reported to promote autophagy in multiple manners, including the AKT1–mTOR–ULK1/2 signaling pathway^22^, ATG9a trafficking via actin^23^ and biogenesis of ATG16L^+^ vesicles^21^. Herein, MIR99AHG enhanced autophagy via cytoplastic ANXA2-induced ATG16L^+^ vesicle biogenesis. MIR99AHG bound to cytoplastic ANXA2 through its 5′ stem-loop structure, and the ANXA2-binding domain was vital for the MIR99AHG-induced autophagy, which means that the MIR99AHG promotes autophagy mainly dependent on the combination with ANXA2. Furthermore, mTOR, a well-known oncogenic protein and autophagy suppressor, was validated to be the target gene of miR-99a^47^. Therefore, our data indicated that MIR99AHG could promote autophagy by binding to ANXA2 as well as generating miR-99a-targeting mTOR.

To our knowledge, this represents the first systematic analysis of the role of *MIR99AHG* gene in LUAD. In summary, our findings provide a new potential theory for the development and progression of LUAD, while the function of MIR99AHG and miR-99a might provide new insight into LUAD diagnosis and treatment.

## Materials and methods

### Patient tissue samples and cell culture

LUAD tissues and paired nontumor tissues were obtained from patients undergoing curative cancer surgery from 2014 to 2019 at the Department of Thoracic Surgery, Jiangsu Cancer Hospital (Nanjing, China). None of the patients included in this study received any preoperative radiation or chemotherapy. All tumors and paired normal tissues were confirmed by experienced pathologists. TNM staging was performed according to the postoperative pathology of the patients. T describes the size of the original tumor; N describes nearby lymph nodes that are involved; M describes distant metastasis. The resected specimens were frozen in liquid nitrogen and then stored at −80 °C until use. Written informed consent was obtained from subjects or their authorized representatives. The study protocol was approved by the Ethics Committee of Nanjing Medical University Affiliated Cancer Hospital and was performed according to the regulation of the Ethics Committee of Nanjing Medical University. This study was approved by the Nanjing Medical University.

All lung cancer cell lines [A549, H1299, SPCA-1, PC-9, H1975, and human bronchial epithelial cell (HBE)] were purchased from Shanghai Institutes for Biological Science (Shanghai, China) and were tested routinely for mycoplasma (last tested 2021.01). A549 cells were cultured in F-12K medium (Gibco); H1299 cells were cultured in RPMI 1640 medium (Gibco, USA); and SPC-A1, PC9, and HBE cells were cultured in DMEM medium (Nanjing, KeyGene), supplemented with 10% FBS and 1% antibiotic–antimycotic agents and cultured at 37 °C in a 5% CO_2_ cell culture incubator. Cells were grown for no more than 25 passages in total for any experiment. All cells were tested negative for mycoplasma contamination using MycoBlue^TM^ mycoplasma detector (Vazyme, China).

### TMA and CISH

A TMA was constructed as described previously^48^. Seventy-four pairs of lung cancer tissues and adjacent normal tissues were used to construct the TMA. RNA CISH was performed to detect MIR99AHG expression in TMA using a digoxigenin-labeled probe as described previously^48^. The sequence of the MIR99AHG probe is listed in Supplementary Table 5. The results of CISH were scored by two independent observers blind to study design.

### Western blot analysis and antibodies

Protein was extracted from transfected cells as previously described^49^ using 10% or 12% polyacrylamide gradient SDS gel. All antibodies are listed in Supplementary Table 5. For analyses of autophagic flux, H1299 cells after transfection were starved for 6 h in the absence or presence of CQ (100 μM) which inhibits the transformation of autophagosomes into autolysosomes.

### Plasmid constructs, siRNAs, and transfection of cell lines

The full-length cDNA of human lncRNA MIR99AHG, anti-sense of MIR99AHG, and short-hairpin RNAs targeting MIR99AHG were synthesized and cloned into the expression vector pCDNA3.1. The final constructs were verified by sequencing. The sequences of wild-type or mutant mTOR-3′-UTR were synthesized and cloned into the pmirGL0-basic vector. Plasmid vectors were enriched by using the EndoFree Plasmid Maxi Kit (Qiagen). All these vectors and siRNAs for ANXA2 were produced by Realgene Biotechnology (Nanjing, China). The miRNA mimics and inhibitors were provided by RiboBio (Guangzhou, China). The sh-RNA and siRNA sequences are listed in Supplementary Table 5. siRNA and plasmid vector transfection was performed as described previously^48^.

### RNA extraction, gDNA extraction, PCR, and qRT-PCR analyses

RNA extraction, gDNA extraction, and qTRT-PCR were all performed as described previously^48^. Platinum™ SuperFi II PCR Master Mix (Invitrogen) was used to get the PCR product for RNA pull-down assays. Expression of mRNAs or lncRNAs was normalized to the expression of β-actin or GAPDH. The expression of miRNAs was normalized to the expression of U6. The expression of gDNA was normalized to *TPTE*. Primers for miRNAs were provided by RiboBio (Guangzhou, China). The rest of the primers are listed in Supplementary Table 5.

### Cell proliferation and apoptosis assays

Real-time xCELLigence analysis system proliferation assays were performed according to the manufacturer’s instructions (ACEA Biosciences^50^). Colony formation assays and EdU assays were performed as described previously^48^. A flow cytometer (FACScan; BD Biosciences) equipped with CellQuest software (BD Biosciences) was used to detect the cell apoptosis level.

### Cell migration and invasion assays

For both transwell and Matrigel assays, 4*10^4^ transfected cells suspended in proper medium without FBS were plated in the upper chamber of each transwell assay insert (8-μm pore size, Millipore), and medium containing 10% FBS was added to the lower chamber. After incubating for 24 (transwell assays) or 48 (Matrigel assays) hours, the cells on the filter surface were fixed, stained with crystal violet (Sigma), and photographed. RTCA migration assays were performed according to the manufacturer’s instructions (ACEA Biosciences^50^). For wound-healing assays, the cells after transfection were grown in 10% FBS containing medium in six-well plates. The monolayer was scratched and then incubated in fresh medium once the cells reached 70% density and then photographed. The wounding of the cells was photographed after incubation for 18–24 h.

### Autophagic flux assays

For autophagic flux assays, mRFP-GFP-LC3 lentivirus was purchased from HANBIO (Shanghai, China). H1299 cells infected with mRFP-GFP-LC3 lentivirus were transfected as indicated, followed by starvation with RPMI 1640 medium without FBS for 6 h, with or without CQ treatment of (100 μM). Cells were then fixed using 4% paraformaldehyde. Autophagy was determined by quantifying the percentage of cells with LC3-positive puncta, counting at least 100 cells in triplicate per condition.

### Transmission electron microscopy

Transfected H1299 cells were fixed in electron microscope fixative (Servicebio, Wuhan, China) for 3 h at 4 °C. Samples were post-fixed in 1.0% aqueous osmium tetroxide (pH 7.4) for 2 h at 4 °C and dehydrated in a series of water/acetone mixtures progressing to 100% acetone. Cells were infiltrated in sequentially increasing concentrations of Embed 812-Araldite (SPI), and embedded in BEEM capsules. Ultrathin sections were stained with uranyl acetate followed by lead citrate, and viewed with an HT7700 transmission electron microscope (HITACHI).

### In vivo assays

BALB/c nude mice (4 weeks old) were randomized into experimental group and maintained in laminar flow cabinets under specific pathogen-free conditions. All operations were carried out according to protocols approved by the Nanjing Medical Experimental Animal Care Commission. For the xenograft tumor model, transfected H1299 cells were harvested from cell culture plates. Approximately 1 × 10^7^ cells were subcutaneously injected into a single flank of each mouse. Tumor growth was examined every week, and tumor volume was calculated using the following equation: *V* = 0.5 × *D* × *d*^2^ (*V*, volume; *D*, longitudinal diameter; *d*, transverse diameter). Six weeks after injection, the mice were euthanized and tumor weights were measured and used for further analysis. For the tail vein metastatic tumor model, transfected H1299 cells were harvested from cell culture plates. Approximately 1 × 10^6^ cells were injected into the tail veins of six mice, which were sacrificed 8 weeks after injection. The lungs were removed and photographed, and visible tumors on the lung surface were counted.

### Immunofluorescence and immunohistochemistry

For immunofluorescent staining, H1299 cells were fixed in 4% paraformaldehyde in PBS for 15 min, permeabilized with 0.5% Triton X-100 for 10 min, and stained with appropriate antibodies, followed by confocal microscopic analysis. Primary antibodies were against ANXA2 (CST) and ATG16L (Proteintech). Secondary antibodies included Alexa Fluor 488 and 555-conjugated goat anti-mouse or anti-rabbit IgG (Life technologies). Nuclei were stained with DAPI (KeyGene). For IHC staining, serial sections from tumor tissues of nude mouse xenografts were deparaffinized and antigen retrieval was performed using target antigen retrieval solution (Dako). Sections were then incubated with a primary antibody against LC3B (CST) overnight at room temperature. Then sections were incubated with Envision System HRP-labeled polymer anti-rabbit secondary antibodies (Dako). The results of IHC were scored by two independent observers blind to study design.

### FISH assays

FISH assays were performed as described previously^48^. The sequence of the MIR99AHG probe is listed in Supplementary Table 5.

### Subcellular fractionation location

The subcellular localization of ANXA2 and lncRNA MIR99AHG was detected using the PARIS Kit (Invitrogen, Thermo Fisher Scientific) according to the manufacturer’s protocol.

### RNA immunoprecipitation and pull-down assays

RNA immunoprecipitation was performed as described previously^48^, and magnetic beads were conjugated with anti-ANXA2 or control anti-IgG antibody. Biotin RNA Labeling Mix Kit (Roche) and T7 RNA polymerase (Roche) were used to biotin-label and transcribe the full-length as well as truncated fragments of MIR99AHG RNA, using MIR99AHG overexpression plasmid and PCR products of the truncated fragments as a template, respectively. Then, the RNAs were treated with RNase-free DNase I (Promega) and isolated with RNeasy Mini Kit (Qiagen). RNA pull-down assays were performed with a Magnetic RNA-Protein Pull-Down Kit according to the manufacturer’s instructions. After elution of lncRNA-interacting proteins, they were subjected to mass spectrometric analysis. Liquid chromatography mass spectrometry (LC–MS) experiments were performed with a linear ion trap quadrupole mass spectrometer (Thermo Finnigan) equipped with a microspray source.

### Dual-luciferase reporter assays

Dual-luciferase reporter assays were performed as described previously^48^. The mTOR-binding sites of miR-99a were predicted by TargetScan (http://www.targetscan.org/). Luciferase activity was detected using the Dual-Luciferase Assay Kit (Promega) according to the manufacturer’s instructions.

### Statistical analyses

All statistical analyses were conducted using SPSS 23 software (Abbott Laboratories). Data were expressed using plotting individual value. The normal distribution of the data was tested. The significance of differences between groups was estimated by Student’s *t*-test, *χ*^2^ test, as appropriate. Overall survival rates were calculated by the Kaplan–Meier method with the log-rank test applied for comparison. Survival data were evaluated using univariate and multivariate Cox proportional hazards models. Variables with a value of *P* < 0.05 in univariate analysis were used in subsequent multivariate analysis on the basis of Cox regression analyses. Two-sided *P*-values were calculated, and a probability level of 0.05 was chosen for statistical significance. The sample size for patients and animal model were determined as previously described^48,49^. GraphPad Prism 8 and R software 3.5.1 were used to plot the figures.

## Supplementary information

Supplementary figure and table legends

supplementary figure 1

supplementary figure 2

supplementary figure 3

supplementary figure 4

supplementary figure 5

supplementary figure 6

supplementary figure 7

Supplementary Table 1

Supplementary Table 2

Supplementary Table 3

Supplementary Table 4

Supplementary Table 5
