# Supplementary material for: MIR99AHG is a noncoding tumor suppressor gene in lung adenocarcinoma
Source: Cell Death Dis. 2021 Apr 30;12(5):424. doi: 10.1038/s41419-021-03715-7 (PMC8087685; doi:10.1038/s41419-021-03715-7)
Supplement: Supplementary file 1 — Supplementary figure and table legends [file 41419_2021_3715_MOESM1_ESM.docx]

***MIR99AHG* is a noncoding tumor suppressor gene in lung adenocarcinoma**

**Han et al.**

**Supplementary figure legends**

**Supplementary Fig. 1 let-7c and miR-125b2 are downregulated in LUAD due to the genome copy number deletion of *MIR99AHG* from 21q. a** Expression levels of let-7c (P<0.001) and miR-125b2 (P<0.001) in the TCGA cohort. **b** Expression levels of let-7c (P=0.046) and miR-125b2 (P=0.028) in 30 pairs of LUAD tissues and adjacent normal tissues. **c** CHIPBase site (<http://rna.sysu.edu.cn/chipbase/>) showed that the expression of MIR99AHG is highly correlated with the expression of miR-99a, let-7c and miR-125b2. **d** Correlation between MIA99AHG expression level and let-7c (r=0.556, P<0.001) or miR-125b2 (r=0.543, P<0.001) expression level in the TCGA cohort. **e** Correlation between MIA99AHG expression level and let-7c (r=0.339, P=0.029) or miR-125b2 (r=0.565, P=0.001) expression level in the 30-patient cohort. **f** Expression levels of let-7c and miR-125b2 in LUAD cells, with U6 used as the internal control. **g** Correlation between *MIR99AHG* genomic expression level and let-7c (r=0.551, P=0.002) or miR-125b2 (r=0.590, P<0.001) expression level in the 30-patient cohort. **h** Correlation between *MIR99AHG* genomic expression level and let-7c (r=0.122, P=0.004) or miR-125b2 (r=0.140, P=0.001) expression level in the TCGA cohort. A two-tailed Student’s *t*-test was used for statistical analysis. *P<0.05, **P<0.01, *** P<0.001. Error bars, SEM.

**Supplementary Fig. 2 Correlation between MIR99AHG expression and** [**clinicopathologic**](javascript:;) [**feature**](javascript:;)**s. a**-**c** MIR99AHG expression was downregulated at higher T stage (**a**, P<0.001), N stage (**b**, P<0.001) or TNM stage (**c**, P=0.006) in the TCGA cohort. **d** MIR99AHG expression was downregulated in 72.4% (42 of 58) of paired normal and adjacent lung adenocarcinoma tissues by qRT-PCR. **e**-**g** Expression of MIR99AHG was decreased in patients with higher T stage (**e**, P=0.002), N stage (**f**, P=0.039) or TNM stage (**g**, P=0.014) in the 58-patient cohort. **h** Kaplan-Meier analysis of the correlation between MIR99AHG and overall survival. The data were from GSE31210 (n=226, P=0.0415). **i-j** Kaplan-Meier analysis of the overall survival in the TCGA cohort based on LINC00315 (**i**, HR=0.892, P=0.444) and LINC01671 (**j**, HR=0.815, P=0.169). Error bars, SEM. A two-tailed Student’s *t*-test was used for statistical analysis.

**Supplementary Fig. 3 MIR99AHG inhibits the proliferation, migration and invasion abilities of A549 cells. a** Relative expression of MIR99AHG after transfecting MIR99AHG overexpression vectors into H1299 and A549 cells. **b** qRT-PCR shows that the expression of MIR99AHG was downregulated after the short-hairpin RNAs targeting MIR99AHG were transfected into H1299 cells. **c**-**e** MIR99AHG inhibited the proliferation ability of A549 cells, as shown by RTCA proliferation assays (**c**), EdU assays (**d**) and colony formation assays (**e**). **f**-**h** MIR99AHG inhibited migration and invasion abilities of A549 cells shown by RTCA migration assays (**f**), wound-healing assays (**g**), and transwell and Matrigel assays (**h**). All experiments were repeated three times with similar results and images are representative of three independent experiments. A two-tailed Student’s *t*-test was used for statistical analysis. *P<0.05, **P<0.01, *** P<0.001. Error bars, SEM.

**Supplementary Fig. 4 MIR99AHG interacts with ANXA2. a** Representative images of silver staining of protein pulled down by MIR99AHG, and the ANXA2 was identified at 37 kDa. **b** Predicted secondary structure of MIR99AHG with minimum free energy (MFE) method (<http://rna.tbi.univie.ac.at>). The red color indicates strong confidence for the prediction of each base. RNA pull-down detection of the interaction between ANXA2 and MIR99AHG truncations according to the predicated secondary structure. **c** MIR99AHG overexpression did not affect ANXA2 mRNA expression by qRT-PCR. **d** Western blot analysis showed no significant difference in ANXA2 protein expression after overexpression of MIR99AHG. **e** MIR99AHG overexpression did not affect the mRNA expression of autophagy related genes by qRT-PCR. **f** Inhibition efficiency of siRNAs targeting ANXA2 was tested by qRT-PCR. **g** Inhibition efficiency of Si-ANXA2-2 by Western blot. **h-j** Effect of MIR99AHG mutant (Mut-4) lacking the predicted ANXA2-binding domain on autophagy levels in H1299 cells was shown by WB assays (**h**), TEM (**i**) and autophagic flux experiments (**j**). All experiments were repeated three times with similar results and images are representative of three independent experiments. A two-tailed Student’s *t*-test was used for statistical analysis. *P<0.05, **P<0.01, *** P<0.001, n.s. not statistically significant. Error bars, SEM.

**Supplementary Fig. 5 Correlation between let-7c/miR-125b2 expression and clinicopathologic features in LUAD. a** let-7c expression in patients with T2-4 stage LUAD was lower than that in patients with T1 stage LUAD in the TCGA cohort (upper panel, P=0.032), while miR-125b2 expression was independent of T stage (lower panel, P=0.349). **b** let-7c (P=0.323) and miR-125b2 (P=0.115) expression in the TCGA LUAD cohort was not associated with lymphatic matastasis. **c** Expression of let-7c was negatively correlated with TNM stage (P=0.048), while expression of miR-125b2 was not correlated with TNM stage (P=0.056). **d** Kaplan-Meier analyses showed no significant correlation between let-7c (HR=0.757; 95% CI, 0.565-1.016; P=0.063) or miR-125b2 (HR=0.781; 95% CI, 0.583-1.047; P=0.099) and overall survival in TCGA cohort. A two-tailed Student’s *t*-test was used for statistical analysis.

**Supplementary Fig. 6 miR-99a inhibits the proliferation, migration and invasion abilities of LUAD cells. a** Overexpressing MIR99AHG upregulated expression of the miR-99a/let-7c/miR-125b2 cluster in H1299 cells. **b** Overexpression efficiencies of mimics for miR-99a, let-7c and miR-125b2 in H1299 cells. **c** Western blot analysis showed that let-7c and miR-125b did not affect LC3 conversion, or ATG7, p62 and Beclin-1 levels in H1299 cells in the absence (-) or presence (+) of chloroquine treatment. **d** RTCA assays were performed to detect the proliferation ability of H1299 cells transfected as indicated. **e**-**f** miR-99a inhibited the migration and invasion abilities of H1299 cells, as shown by RTCA migration assays (**e**), and wound-healing assays (**f**). **g** Overexpression efficiency of mimics for miR-99a in A549 cells. **h**-**j** miR-99a inhibited the proliferation ability of A549 cells, as shown by RTCA proliferation assays (**h**), EdU assays (**i**) and colony formation assays (**j**). **k**-**m** miR-99a inhibited the migration and invasion abilities of A549 cells, as shown by RTCA migration assays (**k**), wound-healing assays (**l**), and transwell and Matrigel assays (**m**). All experiments were repeated three times with similar results and images are representative of three independent experiments. A two-tailed Student’s *t*-test was used for statistical analysis. *P<0.05, **P<0.01, *** P<0.001, n.s. not statistically significant. Error bars, SEM.

**Supplementary Fig. 7 MiR-99a inhibition partially preserves the biological function of MIR99AHG overexpression.** **a**-**c** Proliferation of transfected H1299 cells as indicated by RTCA proliferation assays (**a**), colony formation assays (**b**) and EdU assays (**c**). **d**-**f** Migration and invasion ability of the indicated H1299 cells was shown by RTCA migration assays (**d**), transwell and Matrigel assays (**e**) and wound-healing assays (**f**). **g**-**h** Autophagy levels of the indicated H1299 cells are shown by WB assays (**g**) and TEM (**h**). All experiments were repeated three times with similar results and images are representative of three independent experiments. A two-tailed Student’s *t*-test was used for statistical analysis. *P<0.05, **P<0.01, *** P<0.001. Error bars, SEM.

**Supplementary tables**

**Supplementary Table 1.** Arm-level copy number deletion related differentially expressed lncRNAs in LUAD.

**Supplementary Table 2.** Correlation between MIR99AHG expression and clinicopathologic characteristics of lung adenocarcinoma (TMA cohort).

**Supplementary Table 3.** Correlation between MIR99AHG expression and clinicopathologic characteristics of lung adenocarcinoma (58-patient cohort).

**Supplementary Table 4.** Univariate and Multivariate Cox-regression analysis of lung cancer survival (TMA cohort).

**Supplementary Table 5.** Primers, probes, siRNAs sequences and antibodies used in this work.
