# Supplementary figures and images for: MIR99AHG is a noncoding tumor suppressor gene in lung adenocarcinoma
Source: Cell Death Dis. 2021 Apr 30;12(5):424. doi: 10.1038/s41419-021-03715-7 (PMC8087685; doi:10.1038/s41419-021-03715-7)

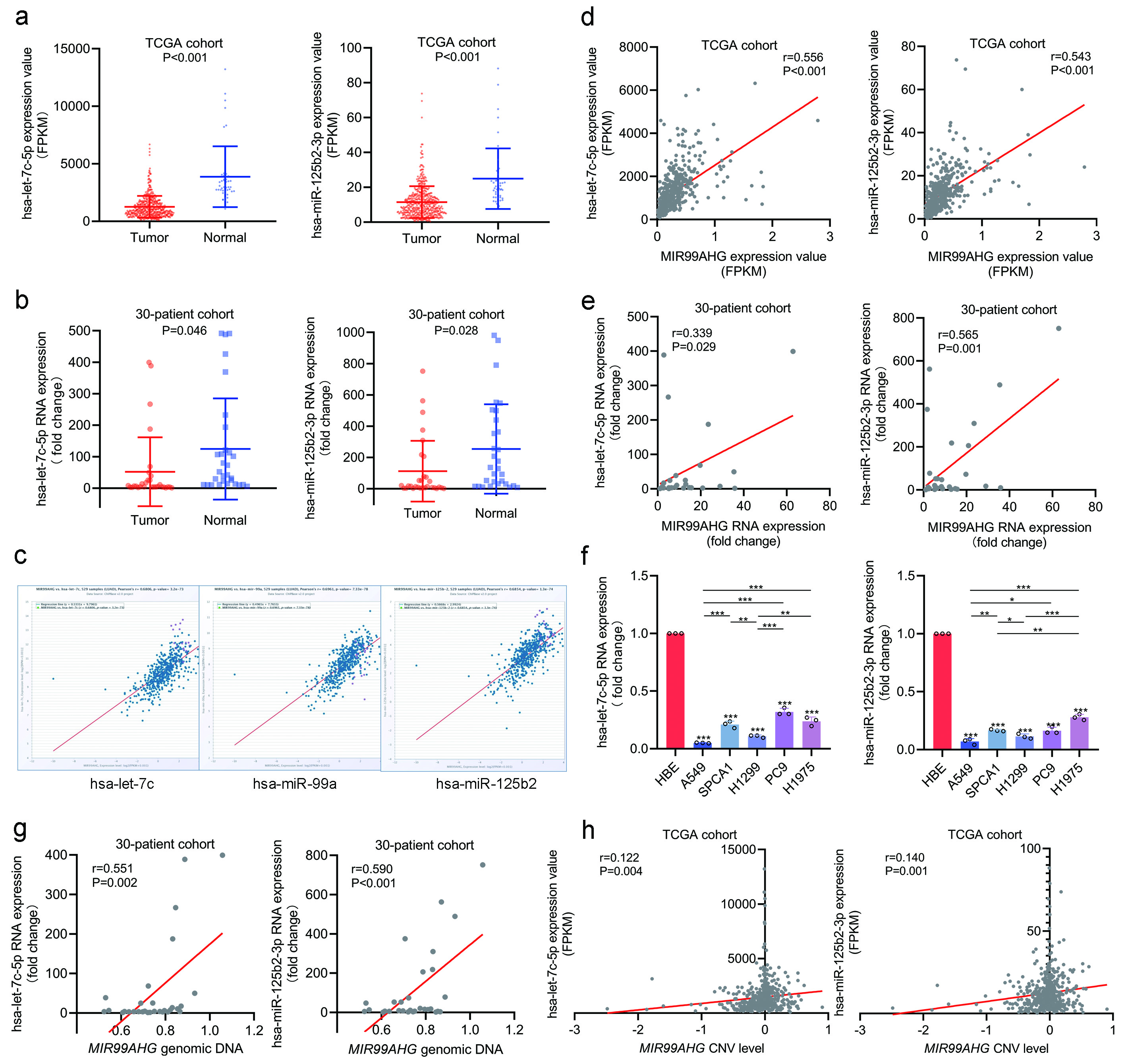

Supplement: Supplementary file 2 — supplementary figure 1 [file 41419_2021_3715_MOESM2_ESM.jpg]

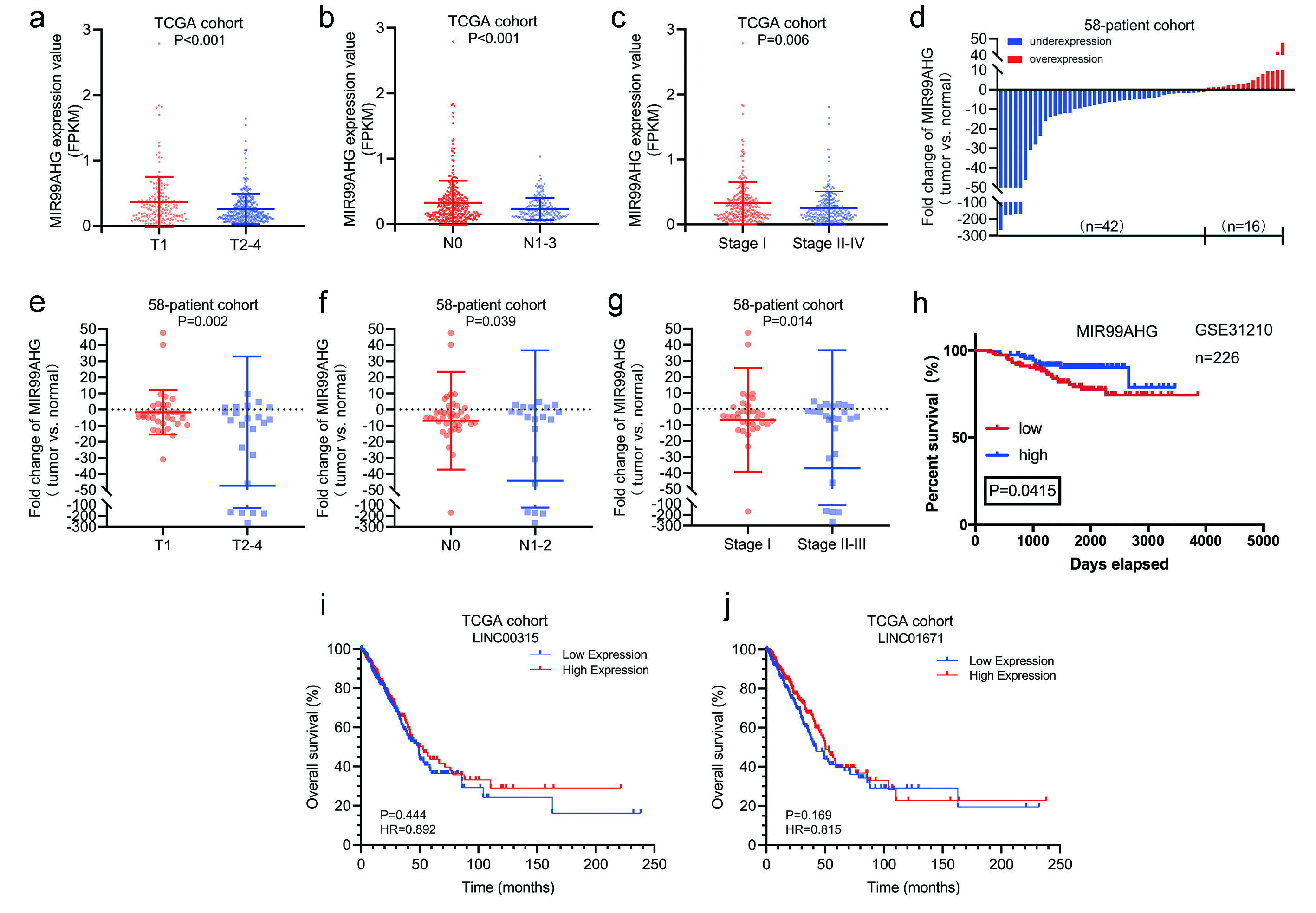

Supplement: Supplementary file 3 — supplementary figure 2 [file 41419_2021_3715_MOESM3_ESM.jpg]

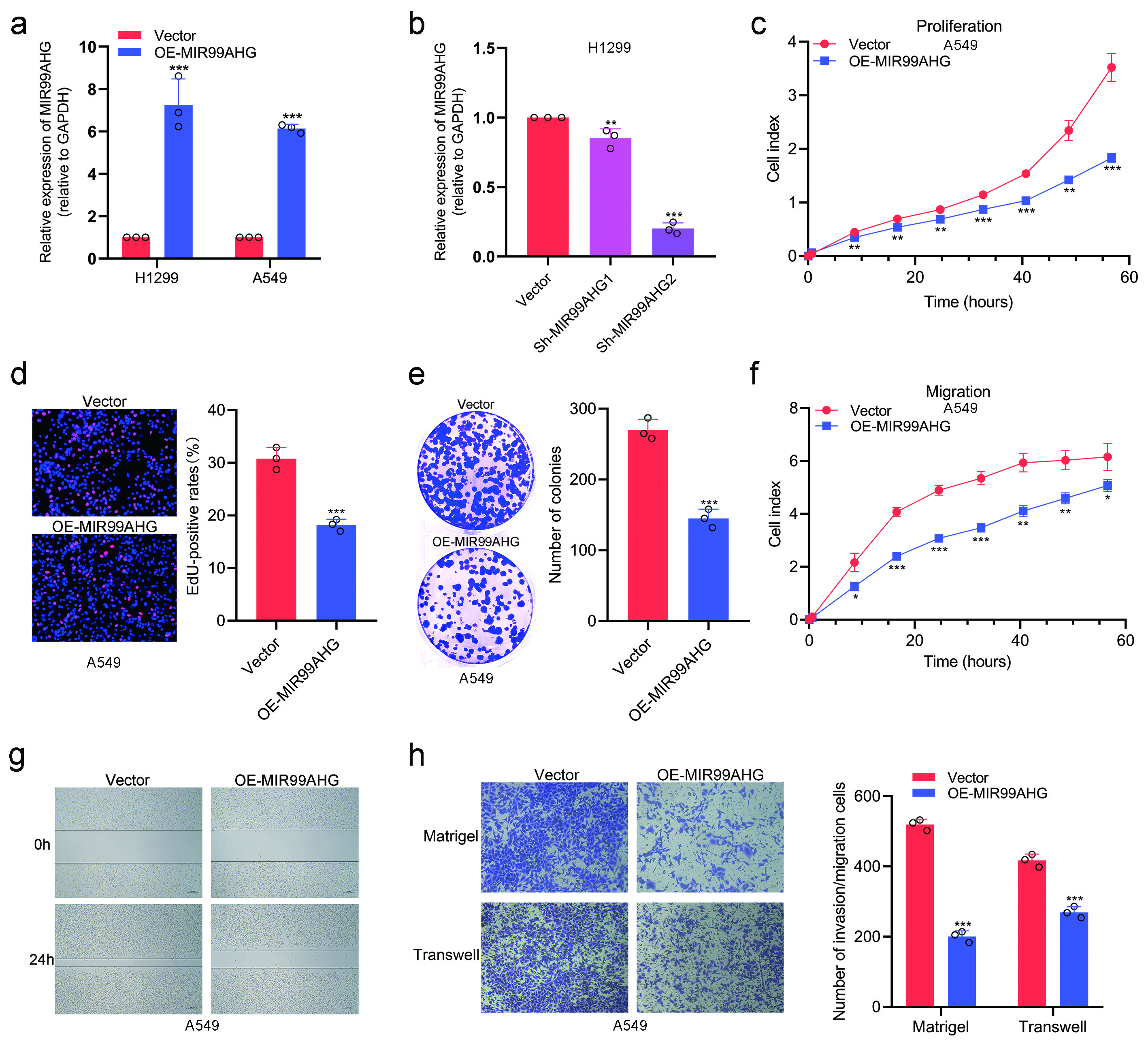

Supplement: Supplementary file 4 — supplementary figure 3 [file 41419_2021_3715_MOESM4_ESM.jpg]

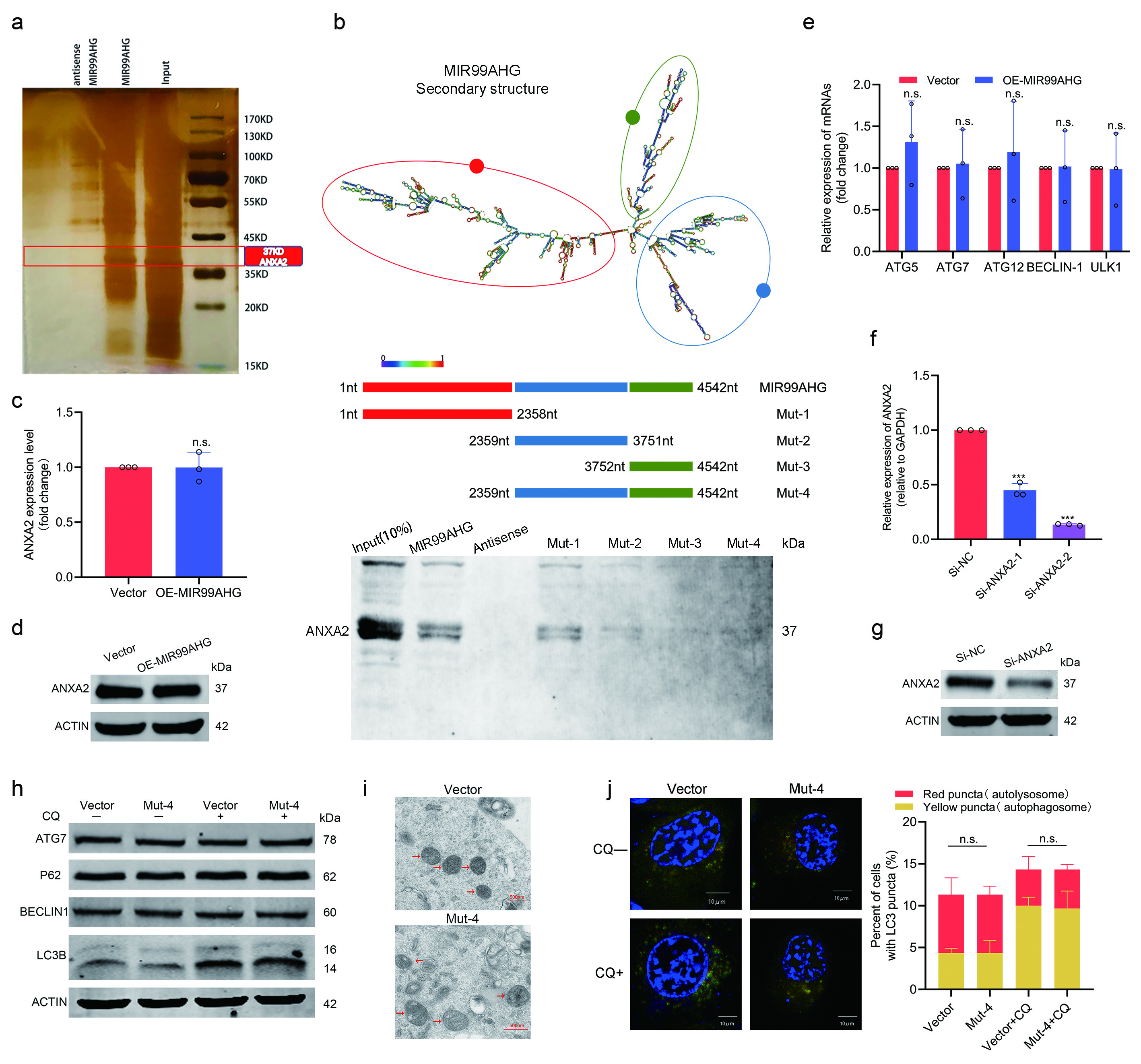

Supplement: Supplementary file 5 — supplementary figure 4 [file 41419_2021_3715_MOESM5_ESM.jpg]

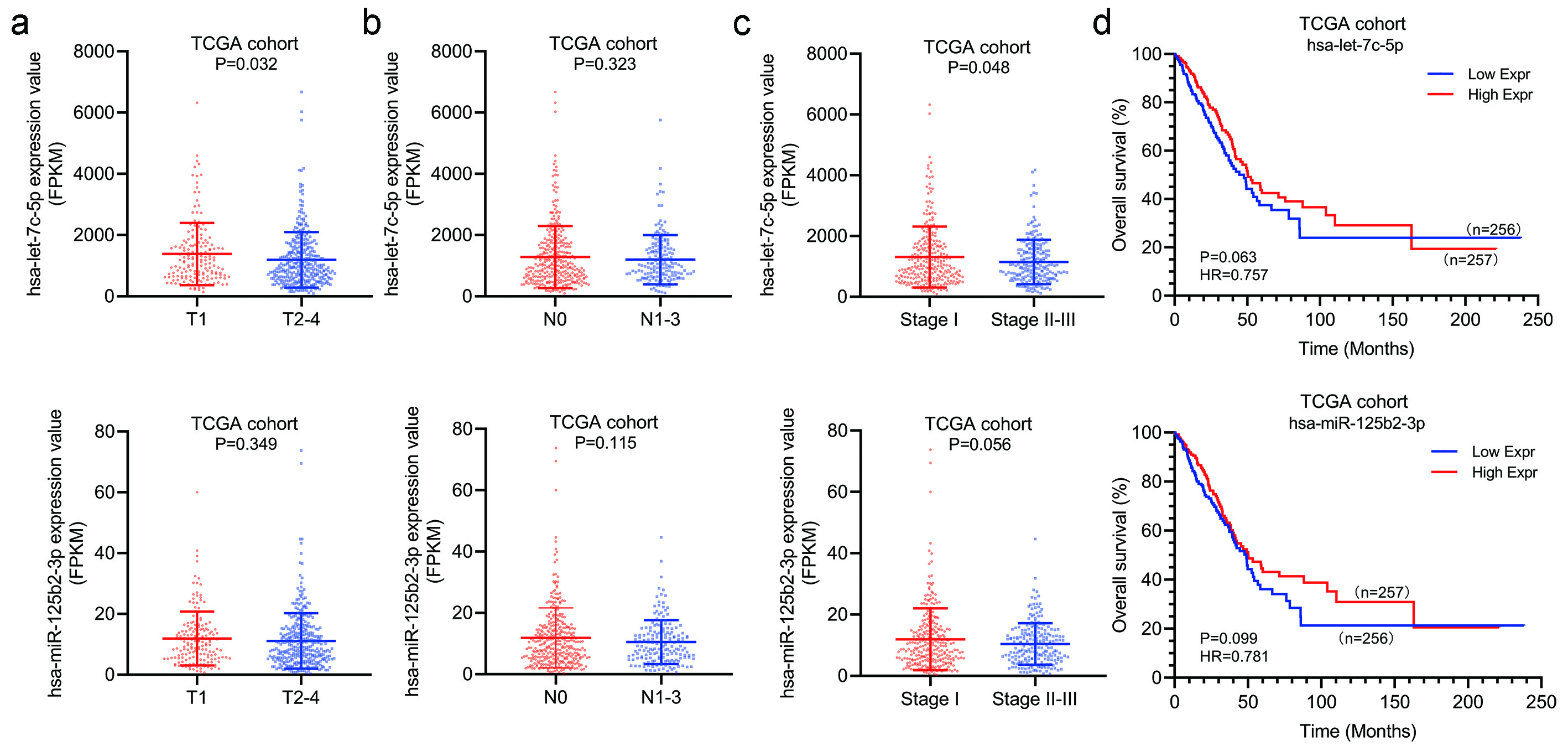

Supplement: Supplementary file 6 — supplementary figure 5 [file 41419_2021_3715_MOESM6_ESM.jpg]

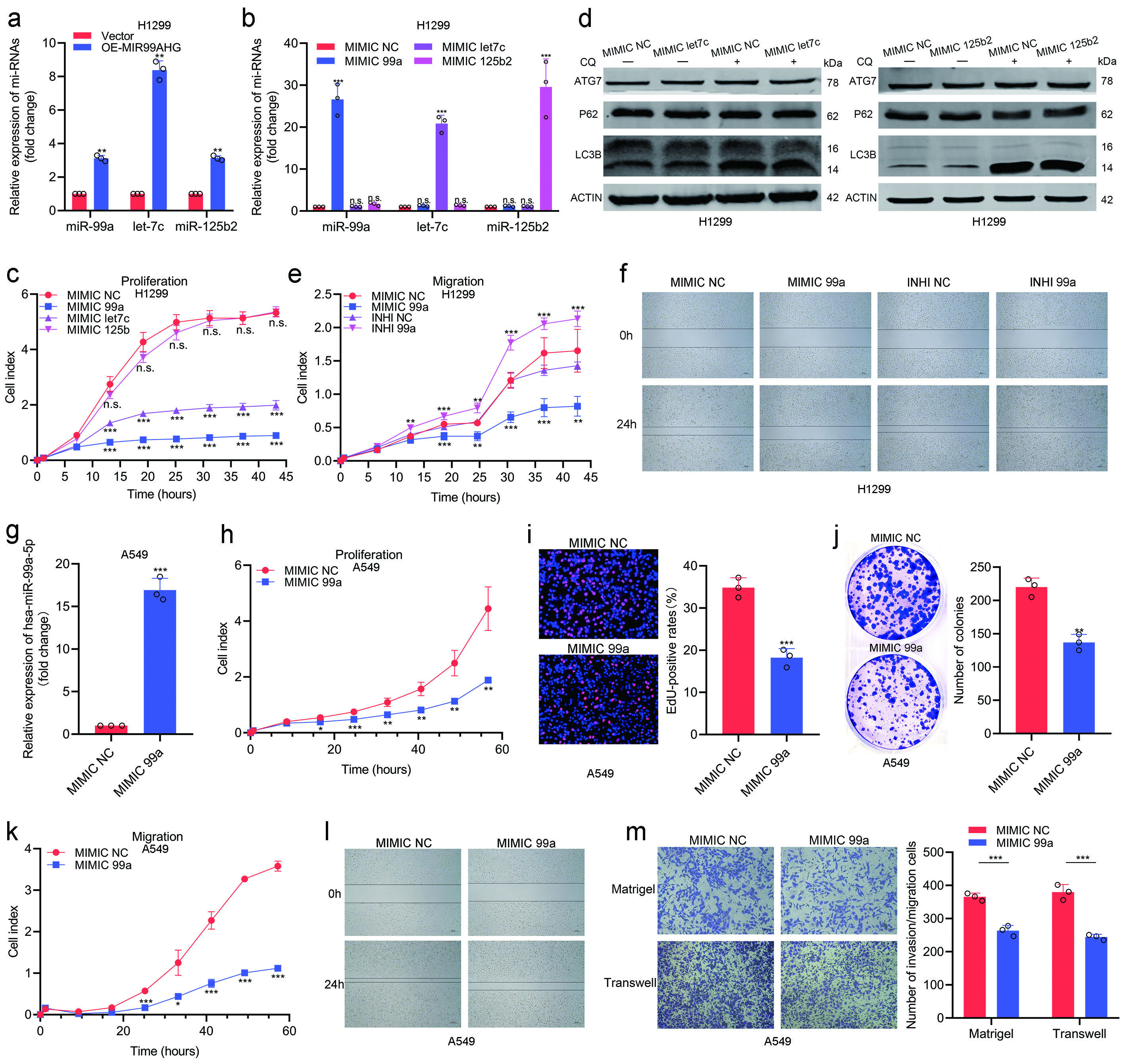

Supplement: Supplementary file 7 — supplementary figure 6 [file 41419_2021_3715_MOESM7_ESM.jpg]

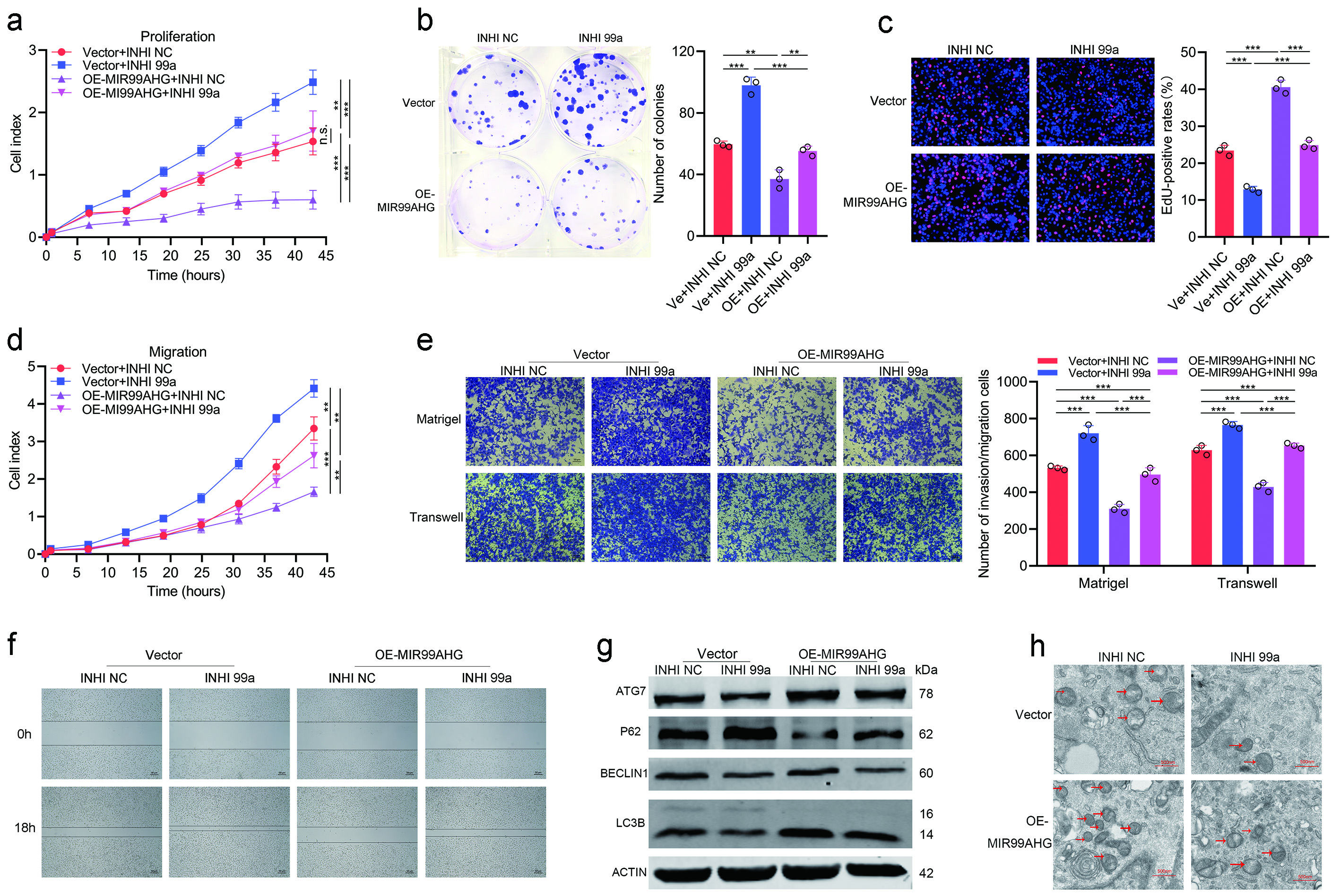

Supplement: Supplementary file 8 — supplementary figure 7 [file 41419_2021_3715_MOESM8_ESM.jpg]
