# Supplementary material for: MIR99AHG is a noncoding tumor suppressor gene in lung adenocarcinoma
Source: Cell Death Dis. 2021 Apr 30;12(5):424. doi: 10.1038/s41419-021-03715-7 (PMC8087685; doi:10.1038/s41419-021-03715-7)
Supplement: Supplementary file 9 — Supplementary Table 1 [file 41419_2021_3715_MOESM9_ESM.docx]

**Supplementary table 1.** Arm-level copy number deletion related differentially expressed lncRNAs in LUAD.

| ENSG NUMBER | cor | p.value | SYMBOL | hg38_SYMBOL | arm-level copy number deletion |
| --- | --- | --- | --- | --- | --- |
| ENSG00000271020 | 0.3602344 | 5.56771E-17 | RP11-10C24.1 |  | 3p |
| ENSG00000271643 | 0.3718299 | 4.54332E-18 | RP11-10C24.3 |  | 3p |
| ENSG00000225873 | 0.1168991 | 0.008420679 | LINC00694 | C3orf86 | 3p |
| ENSG00000271324 | 0.349828 | 4.8442E-16 | RP11-10C24.2 |  | 3p |
| ENSG00000271843 | 0.2403743 | 4.26262E-08 | RP11-245J9.5 |  | 3p |
| ENSG00000235126 | 0.3245914 | 7.45046E-14 | AC128709.3 |  | 3q |
| ENSG00000237167 | 0.212123 | 1.51003E-06 | AC128709.2 |  | 3q |
| ENSG00000273437 | 0.1042647 | 0.018859265 | RP11-434H6.7 |  | 3q |
| ENSG00000243701 | 0.1956727 | 9.0802E-06 | LINC00883 | DUBR | 3q |
| ENSG00000273033 | 0.272363 | 4.49362E-10 | RP11-67L2.2 | LINC02035 | 3q |
| ENSG00000260633 | 0.2188534 | 6.48667E-07 | RP11-375I20.6 |  | 3q |
| ENSG00000273125 | 0.1511382 | 0.00063932 | RP11-115H18.1 | LINC01990 | 3q |
| ENSG00000249364 | 0.1221566 | 0.005885544 | RP11-434D9.1 |  | 5q |
| ENSG00000272416 | 0.1906927 | 1.53995E-05 | CTD-2081C10.7 |  | 5q |
| ENSG00000234292 | 0.2423004 | 3.29734E-08 | RP11-213H15.1 |  | 5q |
| ENSG00000253959 | 0.1494273 | 0.00073744 | CTB-43E15.1 | LINC01863 | 5q |
| ENSG00000271815 | 0.2637588 | 1.62509E-09 | CTD-2235C13.3 |  | 5q |
| ENSG00000272370 | 0.4367963 | 4.91553E-25 | RP11-307L14.1 |  | 5q |
| ENSG00000235172 | 0.1095913 | 0.013549897 | CTB-114C7.3 | LINC01366 | 5q |
| ENSG00000224460 | 0.1080659 | 0.014916079 | RP11-439L18.2 |  | 6q |
| ENSG00000228692 | 0.1592539 | 0.000318198 | RP5-826L7.1 |  | 6q |
| ENSG00000232310 | 0.1145231 | 0.009856891 | RP11-557H15.4 |  | 6q |
| ENSG00000233237 | 0.1389682 | 0.001709144 | LINC00472 | LINC00472 | 6q |
| ENSG00000271967 | 0.2433623 | 2.85941E-08 | RP11-134K13.4 |  | 6q |
| ENSG00000238099 | 0.2318603 | 1.29217E-07 | RP11-12A2.3 | LINC01625 | 6q |
| ENSG00000226032 | 0.1464731 | 0.000940313 | RP1-111C20.3 |  | 6q |
| ENSG00000227508 | 0.1786081 | 5.24804E-05 | RP5-894D12.3 | LINC01624 | 6q |
| ENSG00000273100 | 0.1438802 | 0.00115965 | RP11-302L19.3 |  | 6q |
| ENSG00000270638 | 0.2055344 | 3.06468E-06 | RP3-466P17.1 |  | 6q |
| ENSG00000227502 | 0.2123327 | 1.40467E-06 | RP1-249H1.4 | LINC01268 | 6q |
| ENSG00000231426 | 0.4394966 | 2.33247E-25 | RP5-899B16.1 | FILNC1 | 6q |
| ENSG00000234147 | 0.2190429 | 6.3404E-07 | RP3-460G2.2 |  | 6q |
| ENSG00000272375 | 0.3957389 | 1.86401E-20 | RP11-51J9.6 |  | 8p |
| ENSG00000270076 | 0.157692 | 0.000364886 | AF131215.8 |  | 8p |
| ENSG00000235387 | 0.1066393 | 0.016301831 | LINC00961 | SPAAR | 9p |
| ENSG00000231459 | 0.1201864 | 0.00674165 | LINC00032 |  | 9p |
| ENSG00000272866 | 0.1834797 | 3.23173E-05 | RP11-12D24.10 |  | 9p |
| ENSG00000225434 | 0.1309542 | 0.003135782 | RP11-63P12.6 | LINC01504 | 9q |
| ENSG00000225194 | 0.1867488 | 2.31762E-05 | LINC00092 | LINC00092 | 9q |
| ENSG00000224307 | 0.1653318 | 0.000184536 | RP11-344B5.2 |  | 9q |
| ENSG00000272695 | 0.5502568 | 3.1209E-41 | GAS6-AS2 | GAS6-DT | 13q |
| ENSG00000224511 | 0.1223136 | 0.005821734 | LINC00365 | LINC00365 | 13q |
| ENSG00000230490 | 0.2061408 | 2.86164E-06 | RP11-141M1.3 |  | 13q |
| ENSG00000272143 | 0.1724431 | 9.6726E-05 | FGF14-AS2 | FGF14-AS2 | 13q |
| ENSG00000259504 | 0.1000354 | 0.024287189 | RP11-352D13.5 |  | 15q |
| ENSG00000248079 | 0.1257584 | 0.004569144 | DPH6-AS1 | DPH6-DT | 15q |
| ENSG00000257151 | 0.2156672 | 9.49034E-07 | PWAR6 | PWAR6 | 15q |
| ENSG00000270127 | 0.1373688 | 0.001934159 | RP11-526I2.5 |  | 15q |
| ENSG00000261064 | 0.210508 | 1.82011E-06 | RP11-1000B6.3 | LINC02256 | 15q |
| ENSG00000236914 | 0.1000456 | 0.024272601 | RP11-1008C21.2 | LINC01852 | 15q |
| ENSG00000261863 | 0.1616753 | 0.000256704 | RP11-141J13.5 | LINC01996 | 17p |
| ENSG00000270091 | 0.3368166 | 6.48435E-15 | RP11-78O7.2 |  | 17p |
| ENSG00000186594 | 0.3540195 | 2.04624E-16 | MIR22HG | MIR22HG | 17p |
| ENSG00000262089 | 0.1876238 | 2.11819E-05 | RP11-589P10.5 |  | 17p |
| ENSG00000272799 | 0.1342135 | 0.002459395 | RP11-474N24.6 |  | 18p |
| ENSG00000265752 | 0.1625599 | 0.000237152 | RP11-403A21.1 |  | 18q |
| ENSG00000267414 | 0.362644 | 3.33538E-17 | RP11-456K23.1 |  | 18q |
| ENSG00000266312 | 0.1223153 | 0.005821034 | RP11-111H3.3 | LINC01927 | 18q |
| ENSG00000267325 | 0.1943993 | 1.04068E-05 | RP11-397A16.2 | LINC01415 | 18q |
| ENSG00000268087 | 0.2933446 | 1.60999E-11 | CTC-429P9.2 |  | 19p |
| ENSG00000197332 | 0.2272313 | 2.32018E-07 | ZNF833P |  | 19p |
| ENSG00000267100 | 0.2670909 | 9.93168E-10 | ILF3-AS1 | ILF3-DT | 19p |
| ENSG00000267530 | 0.2088122 | 2.11069E-06 | AC006273.5 | LINC01836 | 19p |
| ENSG00000267519 | 0.1782211 | 5.45119E-05 | MIR24-2 |  | 19p |
| ENSG00000268555 | 0.381758 | 4.89782E-19 | RP11-678G14.3 |  | 19p |
| ENSG00000197813 | 0.1270324 | 0.00417141 | CTC-301O7.4 |  | 19q |
| ENSG00000268355 | 0.1213631 | 0.00621784 | AC006129.1 |  | 19q |
| ENSG00000215386 | 0.1132254 | 0.010729901 | LINC00478 | MIR99AHG | 21q |
| ENSG00000184274 | 0.1419303 | 0.001479689 | LINC00315 | LINC00315 | 21q |
| ENSG00000225431 | 0.2449552 | 2.30623E-08 | AP001626.1 | LINC01671 | 21q |
