# Supplementary material for: MIR99AHG is a noncoding tumor suppressor gene in lung adenocarcinoma
Source: Cell Death Dis. 2021 Apr 30;12(5):424. doi: 10.1038/s41419-021-03715-7 (PMC8087685; doi:10.1038/s41419-021-03715-7)
Supplement: Supplementary file 10 — Supplementary Table 2 [file 41419_2021_3715_MOESM10_ESM.docx]

**Supplementary table 2.** Correlation between MIR99AHG expression and clinicopathologic characteristics of lung adenocarcinoma (TMA Cohort).

| **Characteristics** |  | **MIR99AHG expression** | | **Pearson χ2** | ***P* value** |
| --- | --- | --- | --- | --- | --- |
|  |  | **Low** | **High** |  |  |
| Age | <=65 | 23 | 24 | 0.058 | 0.809 |
|  | >65 | 14 | 13 |  |  |
| Gender | male | 28 | 27 | 0.071 | 0.790 |
|  | female | 9 | 10 |  |  |
| Smoking | yes | 17 | 18 | 0.054 | 0.816 |
|  | no | 20 | 19 |  |  |
| Differentiation | low | 27 | 21 | 2.135 | 0.144 |
|  | middle and high | 10 | 16 |  |  |
| T stage | T1-2 | 29 | 36 | 6.198 | **0.013*** |
|  | T3-4 | 8 | 1 |  |  |
| N stage | N0 | 12 | 29 | 15.806 | **<0.001***** |
|  | N1-2 | 25 | 8 |  |  |
| TNM stage | I | 6 | 24 | 18.164 | **<0.001***** |
|  | II-IV | 31 | 13 |  |  |
| *P* < 0.05 was considered as significant | |  |  |  |  |
