# Supplementary material for: MIR99AHG is a noncoding tumor suppressor gene in lung adenocarcinoma
Source: Cell Death Dis. 2021 Apr 30;12(5):424. doi: 10.1038/s41419-021-03715-7 (PMC8087685; doi:10.1038/s41419-021-03715-7)
Supplement: Supplementary file 11 — Supplementary Table 3 [file 41419_2021_3715_MOESM11_ESM.docx]

**Supplementary table 3.** Correlation between MIR99AHG expression and clinicopathologic characteristics of lung adenocarcinoma (58-patient Cohort).

| **Characteristics** |  | **MIR99AHG expression** | | | **Pearson χ2** | ***P* value** |
| --- | --- | --- | --- | --- | --- | --- |
|  |  | **Low** | | **High** |  |  |
| Age | <=60 | 10 | | 13 | 1.523 | 0.217 |
|  | >60 | 21 | | 14 |  |  |
| Gender | male | 9 | | 14 | 3.140 | 0.076 |
|  | female | 22 | | 13 |  |  |
| Smoking | yes | 15 | | 10 | 0.758 | 0.384 |
|  | no | 16 | | 17 |  |  |
| Differentiation | low | 14 | | 15 | 0.624 | 0.430 |
|  | high | 17 | | 12 |  |  |
| T stage | T1 | 13 | | 7 | 1.637 | 0.201 |
|  | T2-4 | 18 | | 20 |  |  |
| N stage | N0 | 7 | | 16 | 8.133 | **0.004**** |
|  | N1-2 | 24 | | 11 |  |  |
| TNM stage | I | 9 | | 15 | 4.185 | **0.041*** |
|  | II-III | 22 | | 12 |  |  |
| *P* < 0.05 was considered as significant | | |  |  |  |  |
