# Supplementary material for: MIR99AHG is a noncoding tumor suppressor gene in lung adenocarcinoma
Source: Cell Death Dis. 2021 Apr 30;12(5):424. doi: 10.1038/s41419-021-03715-7 (PMC8087685; doi:10.1038/s41419-021-03715-7)
Supplement: Supplementary file 12 — Supplementary Table 4 [file 41419_2021_3715_MOESM12_ESM.docx]

**Supplementary table 4.** Univariate and multivariate Cox-regression analysis of lung cancer survival (TMA Cohort).

|  | Univariate Cox Regression | |  | Multivariate Cox Regression | |
| --- | --- | --- | --- | --- | --- |
| Variable | Hazard Ratio (95% CI) | *p* |  | Hazard Ratio (95% CI) | *p* |
| Age, years |  |  |  |  |  |
| <=65 | 1.00 (reference) |  |  | 1.00 (reference) |  |
| >65 | 2.159 (1.107 to 4.213) | **0.024*** |  | 2.080 (1.012 to 4.274) | **0.046*** |
| Gender |  |  |  |  |  |
| Female | 1.00 (reference) |  |  | 1.00 (reference) |  |
| Male | 0.605 (0.262 to 1.395) | 0.238 |  |  |  |
| Smoking |  |  |  |  |  |
| No | 1.00 (reference) |  |  | 1.00 (reference) |  |
| Yes | 0.705 (0.358 to 1.388) | 0.311 |  |  |  |
| Differentiation | |  |  |  |  |
| Low | 1.00 (reference) |  |  | 1.00 (reference) |  |
| High | 1.107 (0.558 to 2.199) | 0.771 |  |  |  |
| T stage |  |  |  |  |  |
| T1-2 | 1.00 (reference) |  |  | 1.00 (reference) |  |
| T3-4 | 2.555 (1.112 to 5.872) | **0.027*** |  | 1.962 (0.685 to 5.621) | 0.210 |
| N stage |  |  |  |  |  |
| N0 | 1.00 (reference) |  |  | 1.00 (reference) |  |
| N1-2 | 2.323 (1.188 to 4.556) | **0.014*** |  | 2.390 (0.703 to 8.129) | 0.163 |
| TNM stage |  |  |  |  | 0.115 |
| I | 1.00 (reference) |  |  | 1.00 (reference) |  |
| II | 3.467 (1.468 to 8.189) | **0.005**** |  | 1.644 (0.154 to 17.549) | 0.681 |
| III | 2.571 (1.033 to 6.400) | **0.042*** |  | 2.599 (0.277 to 24.369) | 0.403 |
| FAM83H-AS1 | |  |  |  |  |
| Low | 1.00 (reference) |  |  | 1.00 (reference) |  |
| High | 0.329 (0.160 to 0.674) | **0.002**** |  | 0.354 (0.155 to 0.805) | **0.013*** |
| *P* < 0.05 was considered as significant | |  |  |  |  |
