# Supplementary material for: MIR99AHG is a noncoding tumor suppressor gene in lung adenocarcinoma
Source: Cell Death Dis. 2021 Apr 30;12(5):424. doi: 10.1038/s41419-021-03715-7 (PMC8087685; doi:10.1038/s41419-021-03715-7)
Supplement: Supplementary file 13 — Supplementary Table 5 [file 41419_2021_3715_MOESM13_ESM.docx]

**Supplementary table 5.** Primers, probes, siRNAs sequences and antibodies used in this work.

| Primers | Forward Primer |
| --- | --- |
| MIR99AHG-F | 5'-AAGAGCTAGCAGCCAATCCG-3' |
| MIR99AHG-R | 5'-TGCCTTTGAGGTGCTCTTGT-3' |
| ANXA2-F | 5'-TCTACTGTTCACGAAATCCTGTG-3' |
| ANXA2-R | 5'-AGTATAGGCTTTGACAGACCCAT-3' |
| mTOR-F | 5'-GCAGATTTGCCAACTATCTTCGG-3' |
| mTOR-R | 5'-CAGCGGTAAAAGTGTCCCCTG-3' |
| ATG5-F | 5'-AAAGATGTGCTTCGAGATGTGT-3' |
| ATG5-R | 5'-CACTTTGTCAGTTACCAACGTCA-3' |
| ATG7-F | 5'-CAGTTTGCCCCTTTTAGTAGTGC-3' |
| ATG7-R | 5'-CCAGCCGATACTCGTTCAGC-3' |
| ATG12-F | 5'-CTGCTGGCGACACCAAGAAA-3' |
| ATG12-R | 5'-CGTGTTCGCTCTACTGCCC-3' |
| BECLIN-1-F | 5'-GGTGTCTCTCGCAGATTCATC-3' |
| BECLIN-1-R | 5'-TCAGTCTTCGGCTGAGGTTCT-3' |
| ULK1-F | 5'-GGCAAGTTCGAGTTCTCCCG-3' |
| ULK1-R | 5'-CGACCTCCAAATCGTGCTTCT-3' |
| MIR99AHG Mut-1-F | 5'-CCAAGCTTC TAATACGACTCACTATAGGGAGATTGTCTAGGGGAGAGCCTGTA-3' |
| MIR99AHG Mut-1-R | 5'-AGAGGATTTGGGGAAGACTCTCTA-3' |
| MIR99AHG Mut-2-F | 5'-CCAAGCTTC TAATACGACTCACTATAGGGAGACAGGCTGTAGTTTGCTGACC-3' |
| MIR99AHG Mut-2-R | 5'-ACCTTATTTCACAGACCAAACACT-3' |
| MIR99AHG Mut-3-F | 5'-CCAAGCTTC TAATACGACTCACTATAGGGAGACTGTTTTGAACACTACAAAAGTGCT-3' |
| MIR99AHG Mut-3-R | 5'-AAATATTTCTGCCTAGCTCAAAGC-3' |
| MIR99AHG Mut-4-F | 5'-CCAAGCTTC TAATACGACTCACTATAGGGAGAGGACCATAGCTCCCTCACCT-3' |
| MIR99AHG Mut-4-R | 5'-TGCCTAGCTCAAAGCTTACAGAA-3' |
| *TPTE*-DNA-F | 5'-TTTTTGGCCTCTCCCGACTG-3' |
| *TPTE*-DNA-R | 5'-GCGGGTGCTCATAATTGGC-3' |
| *MIR99AHG*-DNA-F | 5'-TGGCACTCACCAAGCTACACT-3' |
| *MIR99AHG*-DNA-R | 5'-GATGTCCATAATAGGGCATTTGTGA-3' |
| GAPDH-F | 5′-CGCTCTCTGCTCCTCCTGTTC-3′ |
| GAPDH-R | 5′-ATCCGTTGACTCCGACCTTCAC-3′ |
| ACTIN-F | 5’-GAAATCGTGCGTGACATTAA-3’ |
| ACTIN-R | 5’-AAGGAAGGCTGGAAGAGTG-3’ |

| **siRNAs and shRNAs** | Sense (5'-3') | Antisense (5'-3') |
| --- | --- | --- |
| shMIR99AHG-1 | 5‘-GATTTGCTTCAAACGACAA-3’ | 5‘-TTGTCGTTTGAAGCAAATC-3’ |
| shMIR99AHG-2 | 5‘-GGATAATTATCCAAATAAA-3’ | 5‘-TTTATTTGGATAATTATCC-3’ |
| siANXA2-1 | 5‘-GUUACAGCCCUUAUGACAU-3’ | 5‘-AUGUCAUAAGGGCUGUAAC -3’ |
| siANXA2-2 | 5‘-GAAAGUACGGCAAGUCCCU-3’ | AGGGACUUGCCGUACUUUC |
| **Probes** |  |  |
| CISH probe | 5‘-DIG-AATCCAGGGAGTTAGGAATGAGCGAATG-DIG-3’ | |
| **Antibodies** | Producer | Item No. |
| ANXA2 | Cell Signalling Tech. | 8235S |
| ATG16L1 | Novus | NB110-60928 |
| ATG16L1 | Cell Signalling Tech. | 8089T |
| LC3B | Cell Signalling Tech. | 3868S |
| ATG7 | Cell Signalling Tech. | 8558S |
| BECLIN-1 | Cell Signalling Tech. | 3495S |
| P62 | Cell Signalling Tech. | 88588S |
| MTOR | Cell Signalling Tech. | 2983T |
| ACTIN | ABCAM | ab32575 |
| GAPDH | Cell Signalling Tech. | 2118S |
| H3 | Cell Signalling Tech. | 3638S |
| IgG | Millipore | EZ-Magna RIP Kit |
| Ki67 | Servicebio | GB13030-2 |
